# Drivers and predictions of coral reef carbonate budget trajectories

**DOI:** 10.1098/rspb.2016.2533

**Published:** 2017-01-25

**Authors:** Fraser A. Januchowski-Hartley, Nicholas A. J. Graham, Shaun K. Wilson, Simon Jennings, Chris T. Perry

**Affiliations:** 1 Geography, College of Life and Environmental Sciences, University of Exeter, Exeter, UK; 2 UMR 248 MARBEC/UMR250 ENTROPIE, UM2-CNRS-IRD-IFREMER-UM1, Université Montpellier 2, Montpellier, France; 3 Lancaster Environment Centre, Lancaster University, Lancaster LA1 4YQ, UK; 4 ARC Centre of Excellence for Coral Reef Studies, James Cook University, Queensland 4811, Australia; 5 Department of Parks and Wildlife, Kensington, Perth, Western Australia, Australia; 6 Oceans Institute, University of Western Australia, Crawley, Western Australia, Australia; 7 Centre for Environment, Fisheries and Aquaculture Science, Pakefield Road, Lowestoft NR33 0HT, UK; 8 School of Environmental Sciences, University of East Anglia, Norwich Research Park, Norwich NR4 7TJ, UK

**Keywords:** bioerosion, coral bleaching, carbonate production, parrotfish, regime shifts, Seychelles

## Abstract

Climate change is one of the greatest threats to the long-term maintenance of coral-dominated tropical ecosystems, and has received considerable attention over the past two decades. Coral bleaching and associated mortality events, which are predicted to become more frequent and intense, can alter the balance of different elements that are responsible for coral reef growth and maintenance. The geomorphic impacts of coral mass mortality have received relatively little attention, particularly questions concerning temporal recovery of reef carbonate production and the factors that promote resilience of reef growth potential. Here, we track the biological carbonate budgets of inner Seychelles reefs from 1994 to 2014, spanning the 1998 global bleaching event when these reefs lost more than 90% of coral cover. All 21 reefs had positive budgets in 1994, but in 2005 budgets were predominantly negative. By 2014, carbonate budgets on seven reefs were comparable with 1994, but on all reefs where an ecological regime shift to macroalgal dominance occurred, budgets remained negative through 2014. Reefs with higher massive coral cover, lower macroalgae cover and lower excavating parrotfish biomass in 1994 were more likely to have positive budgets post-bleaching. If mortality of corals from the 2016 bleaching event is as severe as that of 1998, our predictions based on past trends would suggest that six of eight reefs with positive budgets in 2014 would still have positive budgets by 2030. Our results highlight that reef accretion and framework maintenance cannot be assumed from the ecological state alone, and that managers should focus on conserving aspects of coral reefs that support resilient carbonate budgets.

## 1. Introduction

Ocean warming and climate change are considered the greatest threats to the long-term maintenance of coral-dominated tropical ecosystems. For example, elevated sea temperatures have caused major coral bleaching and associated mortality [1], and are predicted to do so with greater intensity and frequency [2,3]. While ecological trajectories post-climatic disturbance (i.e. whether reefs shift to alternative non- or low coral-dominated states or coral cover rebounds) have received substantial attention [4–6], the geological and geomorphologic implications of these events are less well known, particularly from Indian and Pacific Ocean reefs [7]. In particular, we have limited understanding of how reef disturbance events impact upon the accretion potential of coral reefs, and how this develops with time post-disturbance, particularly where reefs have seen changes in the relative abundance of different coral morphologies and genera [5,8–11]. For example, many Caribbean reefs are now dominated by species that have lower calcification rates, which have not historically been drivers of reef accretion [9], while in Kenya, faster-growing corals have failed to recover after the mass mortality of 1998 [5]. Owing to these changes, the fundamental process of coral and reef growth that provides the structural complexity of reefs and underpins their biodiversity, fisheries and coastal protection functions may be substantial reduced [10], and has implications that may not be apparent from measures of total coral cover alone.

The accretion potential and structural maintenance of a coral reef is fundamentally underpinned by that reef's carbonate budget—the balance between the rate of carbonate production and erosion [7]. Much of this is biologically driven [7,12–15], with carbonate production consisting of skeletal carbonate precipitated by corals and other benthic species (primarily calcareous algae), and biologically driven erosion (termed ‘bioerosion’) occurring due to feeding and other activities (e.g. boring holes for refuge) of bioeroding species of fish (primarily parrotfish), urchins and endolithic boring species [7,14,15]. Where the budget balance is positive, net carbonate accumulation (and reef accretion) may occur, but where ecological changes result in reduced carbonate production and/or increased bioerosion a net negative (erosional) state may follow (e.g. [11]). Prolonged negative budgets have profound implications for the functioning of reefs through their importance in building and maintaining reef structure. Recent data from the Caribbean have indicated a shift to low average rates of carbonate production and bioerosion [8,9], a consequence of decadal declines in coral cover [16], structural complexity [17,18] and fish abundance [19]. Recent studies indicate that carbonate budgets can be dynamic across relatively short time scales and can respond to site-specific emergence of different dominant coral taxa, which may have very different calcification rates [8,11,20,21]. Such short-term dynamics are known to be driven by rapid ecological change trajectories as demonstrated by empirical pre- and post-disturbance event (e.g. bleaching) studies at individual locations, including substantial declines in reef accretion potential [11,20]. However, longer-term carbonate budget dynamics (e.g. time periods greater than a few years) have only been explored by modelling hypothetical responses to alternate climate change and management scenarios [21].

The islands of the Indian Ocean were perhaps the worst impacted reefs globally by the 1998 bleaching event [22], where a large El Niño event coincided with the Indian Ocean dipole, pooling warm water in the western Indian Ocean [23,24]. In 1998, coral cover in the inner Seychelles declined by more than 90% due to severe and extensive bleaching [22]. The loss of coral led to a spatially variable collapse of reef structural complexity, and associated declines in reef fish community diversity [25]. Since 1998, some Seychelles reefs have transitioned to states of high macroalgae cover, while some have recovered, albeit with altered coral composition [26]. By 2011, 12 of 21 surveyed reefs appeared to be recovering well, whereas nine appeared to be locking into an alternate regime dominated by fleshy macroalgae, and simplified fish functional structure [4].

Here we use 20 years of data collected from across the inner Seychelles, pre- and post- the 1998 global bleaching event, to explore disturbance-driven reef carbonate budget trajectories over an unprecedented spatial and temporal scale. Specifically, we assess changes in rates of biologically driven reef carbonate production and erosion on 21 reefs with different trajectories of ecological recovery from bleaching [4]. We ask the following questions. (i) Are geomorphic (recovery to similar carbonate budget state) and ecological recovery trajectories from bleaching equivalent? (ii) How was geomorphic recovery reflected in the components of reef carbonate budgets (e.g. carbonate production, bioerosion and abundance of producing and eroding guilds)? (iii) Is it possible to identify which factors both pre-bleaching and post-bleaching promote reef geomorphic recovery? (iv) What relevance might these factors have in predicting the persistence of Seychelles reefs in the face of further disturbance?

## 2. Material and methods

### (a) Study area

Twenty-one reefs, equally distributed across three different habitat types (carbonate fringing reefs, granitic rocky reefs and patch reefs), were surveyed across the inner Seychelles Islands in 1994 (pre-bleaching), 2005, 2008, 2011 and 2014. At each reef, eight to sixteen 7 m radius replicate point counts (sites) were conducted along the reef slope parallel to the reef crest, spaced to cover up to 500 m of linear reef front. At each site, underlying substrate type, benthic community composition, parrotfish community composition, water depth and estimates of rugosity (using the 6-point scale of Polunin & Roberts [27]) were recorded (see [4] for details). We surveyed the benthic community structure in all years using visual estimates of major benthic categories and, from 2008, also used a 10 m line-intercept transect. Cover of live hard coral (identified to genus), soft coral, macroalgae, sand, rubble, rock and crustose coralline algae to the nearest 5 cm were quantified. In 2014, we also measured the sizes of individual hard coral colonies, recording both the distance below the tape (linear distance) and the length of the surface of each colony. Visual estimates of benthic cover were used to quickly assess benthic cover across the entire site and have been shown to yield similar values to line-intercept transects [28]. We used the same general gross categories for visual and line-intercept surveys, with the exception that hard coral cover was estimated using colony morphology as a class (branching, encrusting and massive), rather than genera for visual estimates. At each point count parrotfish were recorded by species and to the nearest centimetre total length (*L*) using the methodology of Jennings *et al*. [29]. Biomass (*B*) of parrotfishes at each point count was calculated using published length–weight relationships [30]. We assigned parrotfish species to three groups based on their feeding habits: browsers, excavators and scrapers [15,30]. Excavators (primarily *Chlorurus* spp.) and scrapers (primarily *Scarus* spp.) remove pieces of reef substratum (excavators significantly more than scrapers), and are important bioeroders on coral reefs [7,15]. By contrast, browsers predominantly feed on macroalgae and may play a crucial role in inhibiting or reversing shifts to macroalgae dominance on coral reefs [31,32]. The biomass of parrotfishes in each group was calculated as the sum of the relevant species's biomass (electronic supplementary material, table S3).

### (b) Calculating the carbonate budget

In 2014, estimates of the carbonate budgets (the net balance between biological carbonate production and bioerosion) were calculated at eight 10 m line-intercept transects at each reef, using a simplified version of the ReefBudget approach adapted for use on Indian Ocean reefs [10]. By convention, the term *G* is used to refer to rates of production or erosion, with units of kg CaCO_3_ m^−2^ yr^−1^. The ReefBudget approach uses colony size, simple geometric relationships and genus-specific growth rates (cm yr^−1^) and skeletal densities (g cm^−3^) for hard coral and for crustose coralline algae to calculate annual carbonate production by each colony under the line-intercept, and converts this to a carbonate production rate (see the electronic supplementary material, table S1 for growth rates and SI; see [10] for full details). Mean genera-specific growth and density rates for Indo-Pacific corals were obtained through a review of the literature. Unfortunately, there is limited spatial and temporal data in growth rates for many genera and morphologies globally, let alone on regional scales, particularly in the Indian Ocean [33]. Thus, in order to test the sensitivity to differences in growth rates, we also calculated carbonate production by corals at both the upper and lower 95% confidence intervals (CIs) around the mean of each genera/morphology combination (see the electronic supplementary material), and reran the analysis on 9999 random permutations of the data to produce a distribution of model coefficients (see the electronic supplementary material). While there was some reclassification of reefs as positive or negative in 2014, there were no changes in overall patterns.

Bioerosion of reef substrate was calculated from feeding and erosion rates of parrotfish, the percentage of available substrate accessible to macrobioeroders (e.g. sponges, worms and bivalves) or endolithic bioeroders (e.g. cyanobacteria and fungi), and reef rugosity (see the electronic supplementary material and figure S1). Additionally, we recorded urchin species abundance and test size along a 10 m × 2 m transect at each site in 2014. However, urchins were not sufficiently abundant to impact the overall budget balance at any reef (electronic supplementary material, figure S3). Because of their rarity and because we only had urchin abundance data for 2 years (2008 and 2014) and did not have size data for 2008, we excluded urchin erosion from our comparison between years and analysis.

In 1994 and 2005, coral cover was only recorded to morphological level using visual estimates of benthic cover, and not to genera level. To ensure that estimates of carbonate production from 1994 to 2014 were consistent, we converted all annual visual estimates of benthic cover to carbonate production using relationships between 2014 carbonate production estimates (*G*) and visual estimates of the cover of branching, encrusting, massive and table corals. As crustose coralline algae cover was negligible in all years, we assumed carbonate production was solely a function of coral abundance and thus fitted multiple regression relationships through the origin. Cover of all coral morphological types were significantly related to carbonate production, and linear models using morphological cover were significantly better than using total coral cover alone (electronic supplementary material, table S4). We used the regression parameters to estimate carbonate production from the visual estimates of coral cover, with an assumption that relative contribution of genera to each morphological category remained constant through time. There is evidence that the proportion of *Acropora* branching corals on recovered reefs in 2014 (approx. 70% of branching corals) is lower than suggested by data from 1997 (more than 90% of branching corals) [34], meaning that the reconstructed carbonate production rates in 1994 are probably conservative, but only by a small margin.

### (c) Data analysis

We analysed how carbonate production, bioerosion and ecological groups that contribute to budget states and overall carbonate balance differed between years, and between reefs that recovered or underwent a regime shift following the 1998 bleaching event [4]. To account for significant non-normality and heteroscedasticity in our data, we square-root-transformed the data where appropriate, and used weighted least-squares linear mixed models where each data point was weighted by the reciprocal of the group (year and regime status, *n* = 10) variance, with reef (*n* = 21) as a random factor. Analysis was conducted in R 3.1.1 [35] using the *lmer* function from the package *lme4* and the *lsm* function in the package *lsmeans* for post hoc comparisons [36,37]. Calculating *p-*values in mixed models is problematic due to the null distribution not being *t*-distributed, and therefore differences between groups were assessed using standardized model coefficients and the 95% CIs around the coefficients, where if the 95% CIs do not overlap with zero, it is indicative of a significant result [36]. Browsing parrotfish biomass, macroalgal, branching coral and massive coral cover were zero-inflated, and therefore we used zero-inflated Poisson regression using the *zeroinfl* function in the *pscl* package in R [38], and identified significant interaction effects using a likelihood ratio test.

### (d) Boosted regression trees

We selected several different physical and ecological variables that have been identified as important in carbonate budgets on coral reefs, and which were collected across multiple years, including 1994. We also selected variables that were likely to be influential across these time scales, such as abundance of bioeroders [39] and ecological predictors important in determining regime shifts (biomass of browsing, excavating and scraping parrotfish, cover of macroalgae, branching, massive and encrusting coral, depth, wave exposure, structural complexity and reserve status—see the electronic supplementary material, table S5 for details). To assess which ecological or physical variables pre-bleaching (1994 data) and post-collapse (2005 data) were associated with accreting or eroding reefs 15 years post-bleaching, a boosted regression trees (BRTs) machine learning modelling technique was performed using the gbm.step routine in the *dismo* package [40]. Data for each predictor variable were averaged at the reef level in both 1994 and 2005. We then classified the carbonate budget (*G*) for each site in 2014 as either net positive or net negative, and assumed that the response followed a binomial distribution. Owing to many sites having no macroalgal cover in 1994, we used macroalgal presence–absence data for this year, instead of per cent cover. See the electronic supplementary information for details of model and variable selection.

### (e) Hindcasting and forecasting responses to bleaching events

We used the BRT model generated to predict 2014 reef state from 1994 ecological metrics to predict the likelihood of each reef having a positive carbonate budget in future years if there were further bleaching events. We assume that the bleaching event that occurred in early 2016 would be of similar magnitude to that described in 1998 (early reports suggest high mortality of branching corals, particularly *Acropora*; N. Graham 2016, personal communication), and assess the likelihood of positive budget states on Seychelles reefs in 2030 (thus giving the same potential time of recovery as recorded in our study). To identify a threshold of probability above which reefs were more likely than not to be in a net positive carbonate budget state, we used the *optimal.threshold* model with the *PredPrev = Obs* methods in the *PresenceAbsence* package [41].

## 3. Results

### (a) Carbonate production regimes and ecological metrics

**Figure 1.**
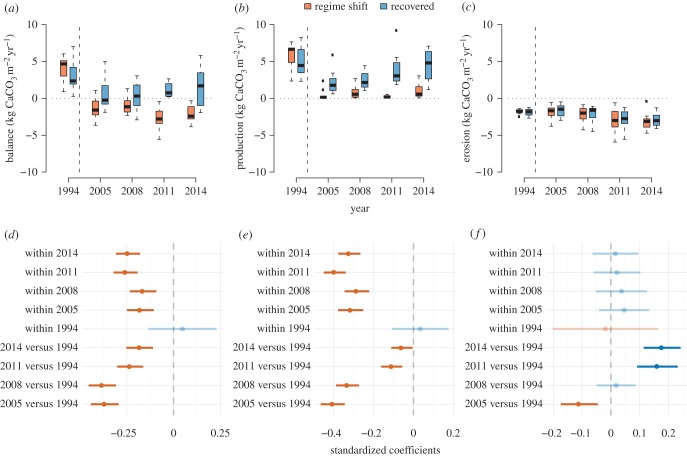
Net and gross carbonate production and erosion rates on Seychelles coral reefs 1994–2014. Box (median and 50% quantile) and whisker (95% quantile) plots and forest plots of model coefficients with 95% CIs of (*a*,*d*) net carbonate budget, (*b*,*e*) gross carbonate production and (*c*,*f*) bioerosion (excluding urchin erosion). Reefs have been divided into reefs considered to have recovered (*n* = 12) or regime-shifted (*n* = 9) after the impacts of the 1998 bleaching event, following Graham *et al*. [4]. Vertical dashed lines on panels (*a*–*c*) indicate the occurrence of the 1998 bleaching event.

All reefs surveyed had positive carbonate budgets in 1994, ranging from only just positive to strongly net positive (mean 3.65*G* ± 0.58). Following the 1994 bleaching, only four reefs out of 21 had a positive budget in 2005, increasing to 8 by 2011 and 2014 (positive budget mean 2014: 2.94*G* ± 0.58; negative budget mean 2014: −1.83*G* ± 0.28, figure 1*a*). Post-bleaching, only reefs that were subsequently classified as recovered by Graham *et al*. [4] showed a positive budget, although not all recovered reefs attained the same pre-bleaching positive budget levels (electronic supplementary material, table S6). By 2014, recovered reefs showed similar levels of net carbonate budgets to recovered reefs in 1994; however, the mixed-effect linear model showed that overall Seychelles reefs' carbonate budgets were lower, with CIs around the model coefficients not overlapping zero (figure 1*d*). Apart from in 1994, recovered reefs had more positive carbonate budgets on average than regime-shifted reefs, with coefficient 95% CI not overlapping zero in any other year (figure 1*a*,*d*).

Overall, model coefficients indicated that carbonate production was lower across both types of reef post-bleaching, and was lower at regime-shifted reefs than recovered reefs within each year, except 1994. Post hoc tests indicated that at recovered reefs carbonate production had regained similar levels to 1994 in 2014 (means of 5.39*G* ± 0.28 and 4.40*G* ± 0.25, respectively; figure 1*b*), but although there was some evidence of increase from 2005 at regime-shifted reefs, this increase was relatively small (2005: 0.50*G* ± 0.10, 2014: 1.02*G* ± 0.171; figure 1*b*). By contrast, bioerosion was greater across all reefs in 2011 and 2014 (mean: −2.96*G* ± 0.19) than in 1994, 2005 and 2008 (mean: 1.90*G* ± 0.11), and there were no apparent differences in total bioerosion between reef states when examining model coefficients (figure 1*c*,*f*).

**Figure 2.**
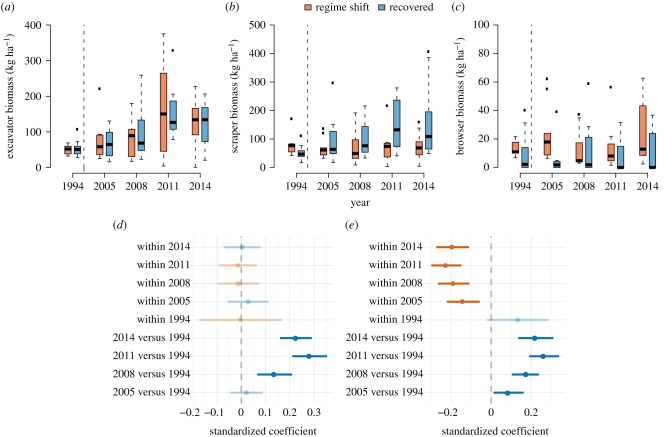
Biomass of parrotfish functional groups. Box (median and 50% quantile) and whisker (95% quantile) plots, and forest plots of model coefficients with 95% CIs of (*a*,*d*) excavating parrotfish, (*b*,*e*) scraping parrotfish and (*c*) browsing parrotfish. Reefs are grouped as figure 1. Note different *y*-axis scales. Vertical dashed lines on panels (*a*–*c*) indicate the occurrence of the 1998 bleaching event. No forest plot is presented for browsing parrotfish due to lack of data to construct a linear model.

Erosion was predominantly the result of parrotfish grazing, a pattern that is reflected in the increase in excavating parrotfish biomass across both reef states with time (figure 2*a*,*d*). Scraping parrotfish biomass, on the other hand, while increasing post-bleaching, primarily increased at recovered reefs, with examination of model coefficients and 95% CI indicating a higher biomass than regime-shifted reefs every sampling year post-bleaching (figure 2*b*,*e*). Browsing parrotfish were generally uncommon on all reefs, although their relative abundance was significantly higher on regime-shifted reefs for all years except 2008 (zero-inflated regression: *z*-value = 6.698, *p* < 0.001; figure 2*c*).

Total coral cover decreased significantly between 1994 and 2005, but increased over time to reach similar levels to 1994 in 2011 and 2014 at recovered, positive status reefs (approx. 30% cover), though little to no change was seen at recovered or regime-shifted negative status reefs from 2005 coral cover (approx. 5%; electronic supplementary material, figure S7*a*). Branched coral showed similar patterns (electronic supplementary material, figure S7*b*). Massive coral cover was lower at regime-shifted sites even before bleaching, and while declining across both reef states, was generally higher at recovered reefs (electronic supplementary material, figure S7*c*). Macroalgae was more abundant at regime-shifted reefs (zero-inflated regression: *z*-value = 2.191, *p* < 0.05), particularly post-bleaching, with many recovered reefs recorded as having no macroalgal cover in every year (electronic supplementary material, figure S7*e*). Structural complexity was also lower at regime-shifted sites in 1994, and post-bleaching across all reefs, although there is evidence for recovered reef structural complexity returning to pre-bleaching levels.

Notably, the reefs that recovered ecologically but still had negative carbonate budgets in 2014 were generally characterized by lower coral cover, and particularly low (less than 2%) massive coral cover in 2014, and were more likely to have macroalgal presence. They also experienced more than 1*G* more bioerosion (3.70*G* ± 0.22 versus 2.48*G* ± 0.30).

### (b) Sensitivity analysis

Reruns of the analysis using the lower and upper 95% CIs for genera level growth rates indicated little difference in the results from using mean growth rates (electronic supplementary material, figure S5). Using the lower 95% CI rates, only six reefs showed a positive carbonate budget, while 10 reefs had a positive budget using the upper 95% CI rates. No regime-shifted reefs had a positive budget under any growth rate.

### (c) Boosted regression trees

#### (i) Pre-bleaching ecological conditions

**Figure 3.**
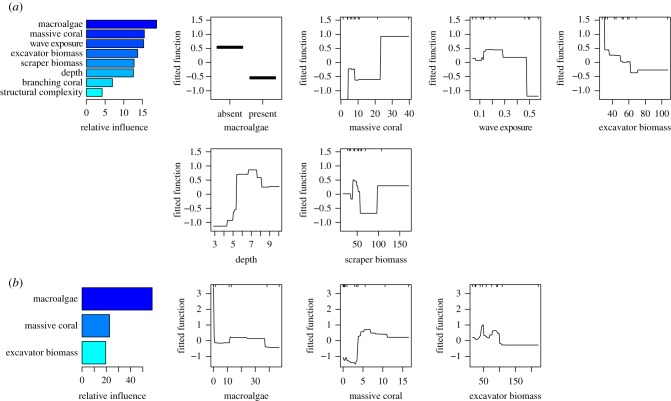
Relative influence (leftmost column) and partial dependency plots (for variables more than 10% relative influence) for the most influential variables in the boosted regression tree analysis for each set of ecological predictors. Predicting from (*a*) pre-bleaching (1994) and (*b*) post-bleaching (2005) ecological conditions. (Online version in colour.)

We found that nine of twelve pre-bleaching predictor variables (browser parrotfish biomass, habitat and reserve status were dropped from the model) had more than 10% relative influence on carbonate budgets (figure 3*a*). Reefs where macroalgae were absent (19% relative influence) had higher cover of massive corals in 1994 (16%), and those deeper than 5 m (13%) were more likely to have positive budgets. In contrast, reefs that are exposed to wave energy greater than 0.25 J m^−3^ (15%) were more likely to have net negative budgets, and there was a negative relationship between excavating parrotfish biomass in 1994 and reef accretion in 2014 (15%). The role of scraping parrotfish biomass (12%) in influencing reef budget state was unclear (figure 3*a*).

#### (ii) Post-collapse conditions

The optimal BRT model based on the post-bleaching data included only three variables, all of which had more than 10% relative influence on the model and were present in the pre-bleaching model (figure 3*b*). Reefs with no or very little (less than 5% cover) macroalgae present in 2005 were considerably more likely to be in a positive budget state (60% relative influence) in 2014. The reefs with positive budgets also had more than 5% massive coral cover (22% relative influence). There was a negative relationship between excavator biomass and budget status (17% relative influence) and reefs with over approximately 90 kg ha^−1^ of excavator biomass were highly likely to have net negative budgets.

### (d) Forecasting response to potential 2016 bleaching

**Figure 4.**
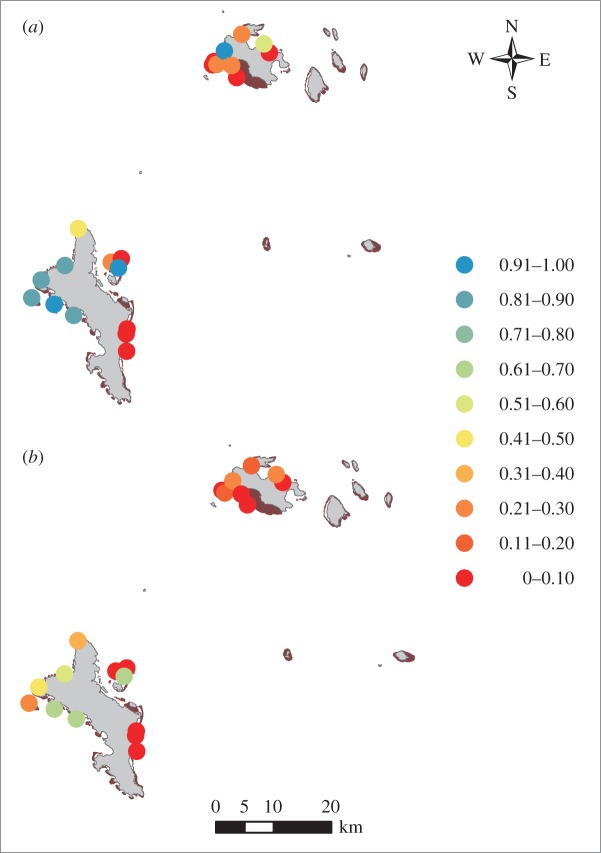
Predicted probabilities of a reef having a net positive (1) or negative (0) carbonate budget. (*a*) Likelihood of having a net positive budget in 2014, predicted using the BRT model based on 1994 data. 95% of these predicted states match with the empirically calculated states. (*b*) Using the same model, likelihood of positive budget states on Seychelles reefs in 2030 if there was a bleaching event in 2016 of similar magnitude to that in 1998 (similar post-bleaching interval as between the 1998 bleaching and 2014 surveys).

When the BRT model based on 1994 data was used to predict the carbonate budget in 2014, eight of 21 reefs were predicted to be in a net positive budget state (predicted probability threshold more than 0.265). For seven of the eight reefs, this prediction was supported by data from 2014 (figure 4*a*). Our model successfully predicted that no reef had a net negative carbonate budget in 2014 when empirical data indicated that the carbonate budget was positive.

When we used the BRT model based on ecological data collected in 2014 to predict the likelihood of each reef having a positive carbonate budget in 2030, only six reefs were predicted to have a net positive budget state (figure 4*b*). Moreover, probabilities of a positive budget on these reefs in 2030 are consistently lower than in 2014. No reef that was regime-shifted in 2014 has a predicted probability of greater than 0.2 so that the reef will be in a net positive budget state in 2030.

## 4. Discussion

Reefs in the Inner Seychelles appear to have followed two divergent carbonate budget trajectories after the 1998 bleaching event that overlap, but are not identical to the trajectories of ecological recovery identified by Graham *et al*. [4]. Of the 21 reefs surveyed, only 8 of the 12 reefs considered ‘recovered’ from an ecological perspective post-bleaching had positive carbonate budgets in 2014, 16 years after the 1998 event. No ‘regime-shifted’ reefs recovered to a positive budget state, despite all reefs having significantly positive budgets (mean > 3.5*G*) pre-bleaching. Additionally, unlike the carbonate budgets measured across the remote Chagos Archipelago, which have mostly recovered to high values [10,42], this study documents far more divergent carbonate budget trajectories over time. Notably, the magnitude of change in average net carbonate budgets across the Seychelles from pre-bleaching (approx. 4*G*) to 2014 (approx. −1.5*G*) levels is significantly greater than that estimated for reefs in the eastern Pacific after previous El Niño-driven disturbance events [11], or in the Caribbean over a similar time period [9], illustrating the extent of damage and subsequent recovery on some Seychelles reefs. However, even at recovered reefs the net balance was substantially lower than on most Chagos reefs [10], with only one reef exhibiting a net budget more than 5*G*, a value considered near the minimum for undisturbed *Acropora*- dominated Indian Ocean fore-reefs [42,43].

In the Caribbean, reduced rates of carbonate production have resulted from both overall declines in coral cover and shifts in coral community assemblages. Faster-growing branching *Acropora* and massive/columnar *Orbicella* spp. have been decimated by disease over the past several decades, with the succession of plating and domed *Agaricia* and *Porites* species leading to a shift towards lower carbonate production potential [8,39]. On Seychelles reefs, there is little evidence that such coral transitions have occurred at reefs that have either positive or negative budgets. Rather, positive carbonate budgets in 2014 are predominantly due to relatively lower mortality of massive corals, and a rebound of branching (primarily *Acropora* spp.) corals to more than 10% cover. By contrast, negative budgets, on both ecologically recovered and regime-shifted reefs, are associated with almost complete loss of massive corals, and relatively low recovery of branched corals. Massive corals tend to be more resistant to bleaching [44], provide persistent reef structure at the seascape scale and, while linear growth rates are low, make a consistent contribution to reef budgets.

Differences between Caribbean and Seychelles carbonate budget states are further attributable to differences in the trajectories of the parrotfish community and how this impacts overall bioerosion between the regions. In the Caribbean, parrotfish populations have declined due to overfishing and a general loss of structural complexity has impacted recruitment [18,45]. By contrast, in the Indian Ocean parrotfish biomass (and therefore erosion) has increased across Seychelles, Chagos [10] and the Maldives [46]. As parrotfishes increase in size, the area of reef they can graze and the volume of material they remove with each bite increases exponentially [15,47,48]. In the Seychelles context, small-bodied parrotfishes that scrape and excavate the reef benthos have declined in abundance, while larger size classes have increased; this situation is unlikely to be stable in the long term due to a lack of replenishment of larger cohorts of fish [49]. This is reflected in the higher rates of bioerosion in more recent years, associated in particular with increased biomass of excavating parrotfishes across all reef states, a situation also observed in the Maldives [46]. Across years, there is an increase in the amount of coral cover above which a net positive carbonate balance is more likely, rising from approximately 11% in 1994 to approximately 18% in 2014, probably directly related to this Seychelles-wide increase in excavating parrotfishes. While scraping parrotfish do show higher biomass at recovered reefs, it is important to note that there was no evidence that scraping parrotfish biomass differed substantially between negative and positive budget reefs within the ecologically recovered cohort. While scraping parrotfishes may play a role by preventing macroalgal expansion on these reefs, the increase in biomass is probably driven by better habitat conditions on recovered reefs (e.g. lower nutrient input [4] and low 1994 macroalgal cover), and it is unclear if there is a mechanistic contribution to the resilience of reef carbonate budgets.

The BRT models indicated that the ecological context of reefs in 1994 did have a substantial bearing on the 2014 budgetary state. In 1994 and 2005, reefs that had a positive budget in 2014 had little to no macroalgae, and higher abundances of more stress-tolerant massive corals that have been shown to recover better from bleaching than competitive branching corals such as *Acropora* [5,44] that previously dominated Seychellois reefs [34]. It is well established that macroalgal blooms can inhibit coral recovery and degrade reef function [26,31], but that macroalgal presence pre-disturbance can impact subsequent carbonate budgets has not previously been made clear. Herbivore biomass was one of the main predictors for ecological recovery in the study of Graham *et al*. [4], emphasizing the importance of controlling macroalgal expansion on recovering reefs. However, the BRT results here indicate that higher biomass of excavating parrotfishes, a nominally herbivore group, made it more likely that reefs would slip into a negative budget state, reflecting the capacity of these fish for bioerosion but their limited ability to remove fleshy macroalgae [15]. While not significantly different between ecological states, excavating parrotfishes were generally at biomasses below the approximately 60 kg ha^−1^ threshold at positive budget state reefs in both 1994 and 2005. The two most significant factors that predicted ecological recovery on Seychelles reefs, depth and structural complexity [4], were of limited importance when considering carbonate budgets. Arguably, this is due to structural complexity being a product of other factors that control carbonate budgets. Much of the value of structural complexity is in the niche space it provides for coral and fish settlement and shelter [17,19,25,49], and since we have direct measures of coral growth forms and scarid abundance, this may have reduced the significance of structural complexity in the final model. The persistence of massive corals may therefore be important beyond their positive contribution to the total carbonate budget; their continued presence is likely to be a good predictor of budget resilience.

The low massive coral cover, the presence of macroalgae and high proportions of branching corals on recovered reefs mirror benthic communities on some reefs in 1994. These reefs subsequently underwent a major regime shift, and are currently in a negative budget state, raising concerns about the long-term consequences of future bleaching on carbonate budgets. Encouragingly, we estimated that if the mortality associated with the 2016 bleaching event is as severe as in 1998, as seems likely, then only two of the eight reefs that currently have a positive budget would shift to a negative budget in 2030 (i.e. recovery potential should be good). However, the fish community structure on these reefs has changed significantly over the past two decades, with reduced cross-scale redundancy among the herbivore community (the ability of different-sized species to compensate for losses) implying that future geomorphic reef trajectories following disturbance may be more uncertain than we predict here [50].

The 1998 El Niño event and subsequent mass coral bleaching and mortality impacted reefs across the world, which have shown disparate levels of recovery to their previous hard coral-dominated state from almost full (e.g. Chagos [42]) to alternative stable states [4]. While some reefs in the Seychelles, where carbonate budgets have recovered post-bleaching, have characteristics that may confer resilience, there is little evidence that other reefs in the Seychelles will ever return to a net positive budget states. Graham *et al*. [4] identified that the rebound of coral reefs in the Seychelles post-bleaching to a coral-dominated state depended primarily on the depth and structural complexity of the reefs. However, identifying the factors that promote geomorphic recovery requires investigation at a higher resolution, potentially due to the complex interactions between coral recruitment, growth, morphology and structural complexity. While ecological recovery of coral reefs and future growth potential are undeniably connected, a third of reefs considered to be ecologically recovering reefs in this study did not show positive carbonate budgets. Our results indicate that massive corals are the mainstay of resilient carbonate budgets, even when branching corals experience almost complete mortality. Measures to reduce potential macroalgal growth (such as controlling run-off) and to ensure the health of massive coral communities (such as banning destructive fishing practices) are essential management tools to promote geomorphic resilience.

## Supplementary Material

Electronic Supplementary Material

## Data accessibility

Details of study sites, including data used for BRTs, can be found in the electronic supplementary material, tables S5–S6.
