# Supplementary material for: Drivers and predictions of coral reef carbonate budget trajectories
Source: Proc Biol Sci. 2017 Jan 25;284(1847):20162533. doi: 10.1098/rspb.2016.2533 (PMC5310043; doi:10.1098/rspb.2016.2533)
Supplement: Electronic Supplementary Material [file rspb20162533supp1.pdf]

SUPPLEMENTARY INFORMATION

1. Carbonate budget calculations

To quantify gross carbonate production and erosion and thus to determine net carbonate production (G, where G = kg CaCO3 m2 yr-1) we used an adaptation of the framework production states approach [1] adapted and parameterised for the Indian Ocean [2]. Carbonate production by corals and coralline algae is calculated using geometric relationships derived from individual colony morphology, rather than calculated using rugosity at the level of the transect; and endolithic bioerosion is calculated using published rates and the proportion of hard substrate under the transect line available for erosion.

Within each point count at each reef in 2014 we measured the distance covered by each category of benthic cover within a 10m line-intercept transect to the nearest 5cm. In contrast to Perry et al. [2], while coral colony size was measured using a chain method (i.e., across the total surface of the colony under the line), this was not applied to other benthic substrate classes. The following groups were recorded: scleractinian corals to the genera and morphological level (e.g., *Acropora* branching, *Porites* massive etc.); crustose coralline algae (CCA); epilithic algal matrix; macroalgae; bare substrate (e.g., granitic rock, limestone pavement); sediment; rubble; and other benthic organisms. We also collected the following in order to characterise the site: (1) gross scale measures of reef topographic complexity; (2) management status; (3) habitat, zone and depth data.

1.1 Calculating Carbonate Production

We used the morphology and size of individual coral colonies in combination with genera specific skeletal density (g cm^{-3}) and linear growth rates (cm $year^{-1}$) across each transect to produce carbonate production rates in kg $CaCO_3$ m^{-2} $year^{-1}$ (where m^2 refers to the planar surface of the reef) – see Table S1 for rates. Where possible, we used published growth rates and skeletal densities from the Western Indian Ocean, but when these were not available means of published growth rates and densities for each coral genera were used. We then used geometric transformations based on colony morphology to give a growth rate for each colony for the area under the transect line (taking a transect line width of 1cm). Massive colonies were assumed to be hemispherical in cross-section, encrusting, foliose and plating colonies, as well as colonies of crustose coralline algae (CCA) were assumed to be growing primarily at the edge of the colony (and at 10% of this growth rate across the remainder of the colony), and for branching colonies, the proportion of the colony area of growing branch tips was assumed to be growing at published rates, and the remainder of the colony at 10% of these rates. The equations used are:

Massive:

$$CP_i = \left(\left(g + \left(\frac{x}{\pi}\right)\right)^2 \pi - \left(\frac{x}{\pi}\right)^2 \pi\right).d$$

Encrusting etc.:

$$CP_i = 2(g.d) + 0.1g.x.d$$

Branching/corymbose etc.:

$$CP_i = (r.c_a.g.d) + (x - c_a.x).0.1g.d$$

Where CP_i = carbonate production for colony *i*, *g* = growth rate, *x* = surface length of colony, *d* = skeletal density and c_a = proportion of colony that are growing axial branches. Measuring the linear surface of growing tips on branching corals is time-consuming, and often impractical. We used data on the size of arborescent, branching, corymbose and digitate colonies and the length of growing tips of each colony across 405 colonies in northern Mozambique (293 Acropora, 62 Pocillopora, 26 Porites, 24 other). We conducted linear regressions between colony

size and length of growing tips for each genera/morphology combination for which we had greater than 20 replicates in order to calculate c_a (see Table S2).

Where CP_j is the total carbonate production of both corals and crustose coralline algae for transect *j* (covering an area of 1000 cm^2 in kg $CaCO_3$ $year^{-1}$). To estimate the production rate of the reef in kg $CaCO_3$ m^{-2} $year^{-1}$, we then multiplied CP_j by 10.

Table S1| Growth rates for genera and morphology combinations used to calculate 2014 carbonate production.

Genera/Taxon	Morphology	Mean Extension rate (cm/yr)	95% CI	Mean Density (g/cm^3)	95% CI	Notes	Refs
Acropora	arborescent	6.931	2.195	1.294	0.373		3-12
Acropora	branching	4.154	1.239	1.340	0.184		3,5,8,13,14
Acropora	corymbose	4.282	1.371	1.340	0.184		5,14,15,16
Acropora	digitate	2.987	1.055	1.227	0.182		3,5,16,17
Acropora	table	8.285	2.825	1.455	0.100		3,16,17,18,19
Astreopora	encrusting, massive	1.029	0.335	1.480	0.240		14,21
Coscinarea	columnar, encrusting, massive	1.785	2.283	1.405	0.062	Average for Siderastredae due to place in phylogentic tree	21,29,36
Cyphastrea	columnar, encrusting, massive	0.532	0.297	1.358	0.294		3,14,36
Echinopora	encrusting, foliose, massive	0.821*	0.274	1.358*	0.132	used Merulinidae average, Faviidae for density	8,14,19,21
Favia	encrusting, massive	0.624	0.189	1.345	0.167		3,14,21,30
Favites	encrusting, massive	0.624*	0.189	1.345*	0.167		3,14,21,30
Fungia	mushroom	0.730	0.411	1.990	0.490		8,16,37
Galaxea	columnar, encrusting, massive	0.900	0.176	1.910	0.529		14
Goniastrea	columnar, encrusting, massive	1.200	0.192	1.726	0.168		3,14,21,36,38,39
Goniopora	columnar, massive	3.360	1.680	1.373*	0.076		14
Leptastrea	encrusting, massive	0.624*	0.189	1.345*	0.167		8
Leptoria	encrusting, massive	0.821*	0.274	1.358*	0.132	used Platygyra averages	14,21,38,40
Leptoseris	encrusting, foliose, plating	1.669	0.200	1.726*	0.168		15
Lobophyllia	encrusting, massive	1.500	0.750	1.370	0.250		14
Merulina	encrusting, plating	1.200	0.192	1.726*	0.168	used the goniastrea averages based on phylogenetic tree similarity	19
Montastrea	columnar, encrusting, massive	0.260	0.020	1.358*	0.132	density average for faviidae	3
Monitopora	branching	3.485	0.593	1.230	0.647	genera average for density	27

Montipora	encrusting	1.625	0.260	1.230	0.647	genera average for density	15
Montipora	foliose, plating	2.985	1.170	1.230	0.647	genera average for density	4,14,15,16,19,41
Pavona	columnar	1.215	0.214	1.726	0.168		14,16,42
Pavona	encrusting	0.335	0.029	1.726	0.168		29,42
Pavona	foliose	2.712	0.283	1.726	0.168		15
Pavona	massive	1.229	0.151	1.726	0.168		15,29,42,43
Platygyra	encrusting, massive	0.874	0.114	1.527	0.462		14,21,38,40
Pocillopora	branching	2.735	0.660	1.400	0.081		3,8,14,16,29,43-48
Pocillopora	submassive	3.536	1.275	1.400	0.081		8,16,21
Porites	branching	2.719	0.401	1.400	0.081		8,9,27,39,41
Porites	columnar	0.568	0.094	1.376*	0.079	density average for poritiidae	31
Porites	encrusting, massive	1.169	0.148	1.372	0.092		3,8,14,20-35
Psammacora	encrusting, massive, plating	1.785	1.648	1.405*	0.062		21,29,36
Stylophora	branching	2.550	0.129	1.405	0.062		31
Turbinaria	foliose, plating	1.983	1.499	1.371	0.066		3,4,14

Table S2| Growing branch tips to colony size ratio

Genera	Morphology	Growing tips: colony size	95% CI	N
Acropora	Arborescent	0.059	0.009	28
	Branching/Corymbose	0.190	0.012	145
	Digitate	0.253	0.016	65
Pocillopora	Branching	0.364	0.027	33
	Submassive	0.338	0.032	17
Porites	Branching	0.221	0.056	21
Stylophora/Stylocoeniella	Branching/submassive	0.327	0.064	10

To calculate the production for a single transect over a year, the following equation was used:

$$CP_j = \sum_{i=1}^{n} CP_1 + CP_2 + \cdots + CP_n$$

1.2 Calculating Reef framework bioerosion

1.2.1 Macro and micro-bioerosion

In general, the macro-borer and endolithic bioerosion communities are less well-characterised in the Indo-Pacific than the Caribbean. This is particularly true of clionaid sponges, which are generally cryptic and difficult to identify in the field, particularly for a non-expert, and are less dominant a part of the macroborer than in the Caribbean. To this end, instead of conducting an intensive search of the substrate for clionaid sponges as described in Perry et al. [1], we utilized published rates of total macrobioerosion in concert with a census of substrate available for bioerosion from the benthic line-intercept transect. This consists of all dead carbonate substrate, rubble and corals. While live corals can prevent settlement of most bioeroding sponges, they are often colonised by other bioeroders. All substrate not available to bioeroders was excluded, and the following equation used:

Microbioerosion = $S*R*E_m$

Macrobioerosion = $S*R*E_a$

Where S is the percentage of surface area of the transect available for erosion, R is the rugosity of the transect, E_m is the erosion rate of microborers (0.055 for shallow sites [49]; 0.258 for reefs >4m depth [50,51], and E_a is the erosion rate of macroborers (0.261 [52]) in kg m^{-2} yr^{-1}.

1.2.2 *Calculating reef rugosity from visual estimates of topography*

In order to calculate macrobioerosion and endolithic bioerosion, the total surface area under the line intercept transect has to be estimated, and the proportion of surface occupied by substrates not susceptible to bioerosion (sand, seagrass, granite etc.) removed. The *Reefbudget* method measures the length of every surface under the transect line, and uses this length divided by the length of the transect line as a complexity conversion factor when calculating the amount of substrate susceptible to either macro or microbioerosion [1]. To convert the visual estimates of reef topography to estimates of complexity for use in calculating macrobioerosion and endolithic microbioerosion, we used the relationship obtained by non-linear least squares regression analysis between the 6-point scale of Polunin and Roberts [53] and measured rugosity (the length of substrate below a 10m transect line) collected by F Januchowski-Hartley across 96 transects in East Africa. The best fitting model was a power relationship of the form:

$$R = e^{b*VT}$$

Where R is the measured rugosity, b is constant equal to 0.29764 and VT is the visual estimate of topography. We estimated the proportion of the variation explained with a pseudo-R^2 that was equal to 1 minus the residual sum of squares of the model divided by the total sum of squares.

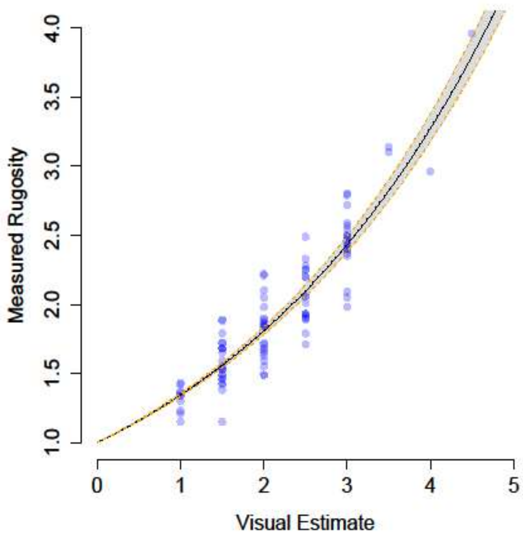

Figure S1| Regression of visual estimates of topography against measured rugosity. R^2=0.85.

1.2.3 *Urchin bioerosion*

The primary agents of echinoid bioerosion belong the family Diadematidae, (*Diadema* spp., and *Echinothrix* spp.) and the genera *Echinometra*, *Echinostrephus* and *eucidaris*. We censused urchin abundance, size and species composition within 10 x 2 m belt transects along the LIT in 2014. We used carbonate ingestion rates from the literature to calculate erosion rate for different sizes of urchins. These census data were combined with carbonate ingestion rates

obtained from published literature. Evaluating the published data on erosion rates against test size across all urchin species suggest a relatively tightly correlated plot. Figure S2A shows the pooled bioerosion rates relative to test size for six species of urchins across 10 studies in the Indian and Pacific Oceans, with Figure S2B showing the rates for *Echinometra mathaei* and the *Diadematidae*. This allows us to calculate the erosion rate (kg urchin^{-1} year^{-1}) for each individual urchin using one of the following equations:

Diadematidae bioerosion (B_D) = $(0.000001*x^{3.4192})*0.365$

Echinometra mathaei bioerosion (B_E) = $(0.0004*x^{1.9786})*0.365$

General equation for all other bioeroding species (B_G) = $(0.0001*x^{2.323})*0.365$

To calculate bioerosion by urchins in kg m^{-2} year^{-1}, we summed the erosion rates of all individual urchins within each transect (U_E), and divided by the surface areas within each transect.

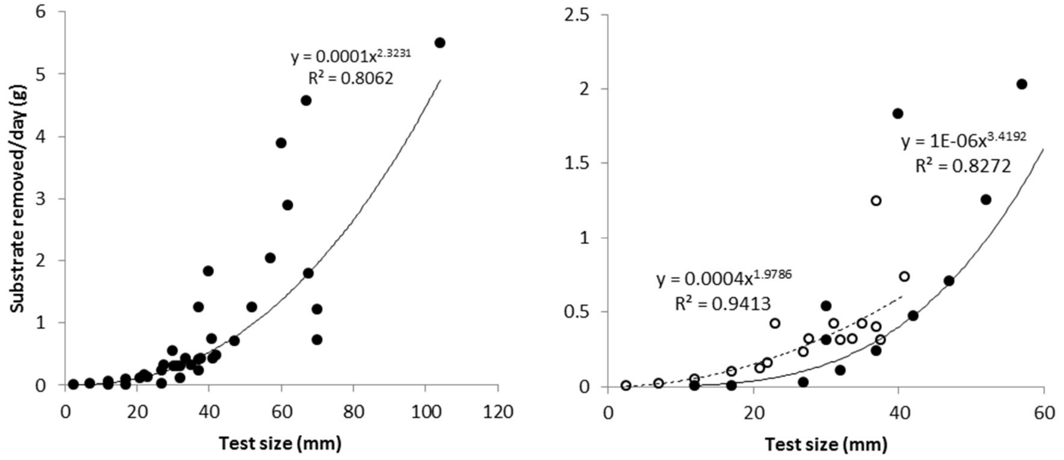

Figure S2| (A) Bioerosion rates (substrate removed/day (g)) for urchins across a range of test size (Indo-Pacific data only). Data aggregated from [refs 54-62]. (B) Bioerosion rates for Diadematidae (closed circles) and Echinometra metra (open circles).

1.2.4 Parrotfish bioerosion

To determine the species-size abundances of parrotfish at each site we used underwater census data for the Seychelles collected in 7m radius point counts by S Jennings (1994) and NAJ Graham (2005-2014). The method we used to calculate bioerosion by fish is based on a model that uses total length to predict bites hr^{-1} for each individual, and then multiplies this by the number of bites leaving feeding scars and the volume of each bite. For species for which data is not available in the published literature, we have either made observations of bite rate in the field, or have used data from similar sized species with the same feeding functional. Daily bite numbers were calculated using diurnal feeding activity reported in Bellwood [63]. Bite volume data has been obtained from published studies [63,64]. Where no bite volume data exists, we have either measured the size of individual bites *in situ* using Vernier callipers to obtain width and length of bite, or obtained from published studies estimates of bite area [e.g., 65]. We have then used a bite depth of 0.1 mm to obtain a conservative estimate of bite volume for *Scarus* sp. Not all bites on the substratum remove material, and we have assumed following Bellwood & Choat [66] that only bites that leave visible scars are eroding the substrate. In order to estimate the proportion of bites resulting in scars on the

substratum for each size class, we extrapolated from published literature [65] and used the same method as above where data was missing. We used the following equation to calculate species specific erosion rates for the median value within each size class:

$$\text{Bioerosion rate (kg.ind}^{-1}\text{yr}^{-1}\text{)} = v.s_{prop}.br.d*365$$

Where v is bite volume (cm^3), s_{prop} is the proportion of bites leaving scars, b_r is bite rate (bites day^{-1}) and d is substratum density (kg cm^{-3}), here taken to be $1.49*10^{-3}$ kg cm^{-3}.

Table S3| Categorisation of parrotfish species into functional group

Browsers	Excavators	Scrapers
Calatomus carolinus	*Cetoscarus bicolor (> 30cm)*	*Scarus caudofasciatus*
Hipposcarus harid	*Chlorurus atrilunula*	*Scarus falcipinnis*
Leptoscarus viagiensis	*Chlorurus caprastroides*	*Scarus ferrugineus*
	Chlorurus japanensis	*Scarus festivus*
	Chlorurus sordidus	*Scarus grenatus*
	Chlorurus strongylocephalus	*Scarus ghobban*
	Scarus rubroviolaceus (TP)	*Scarus globiceps*
		Scarus niger
		Scarus prasiognathus
		Scarus psitticus
		Scarus rubroviolaceus (IP)
		Scarus russellii
		Scarus scaber
		Scarus tricolor
		Scarus viridifucatus

2. Contribution of urchin bioerosion to carbonate budget

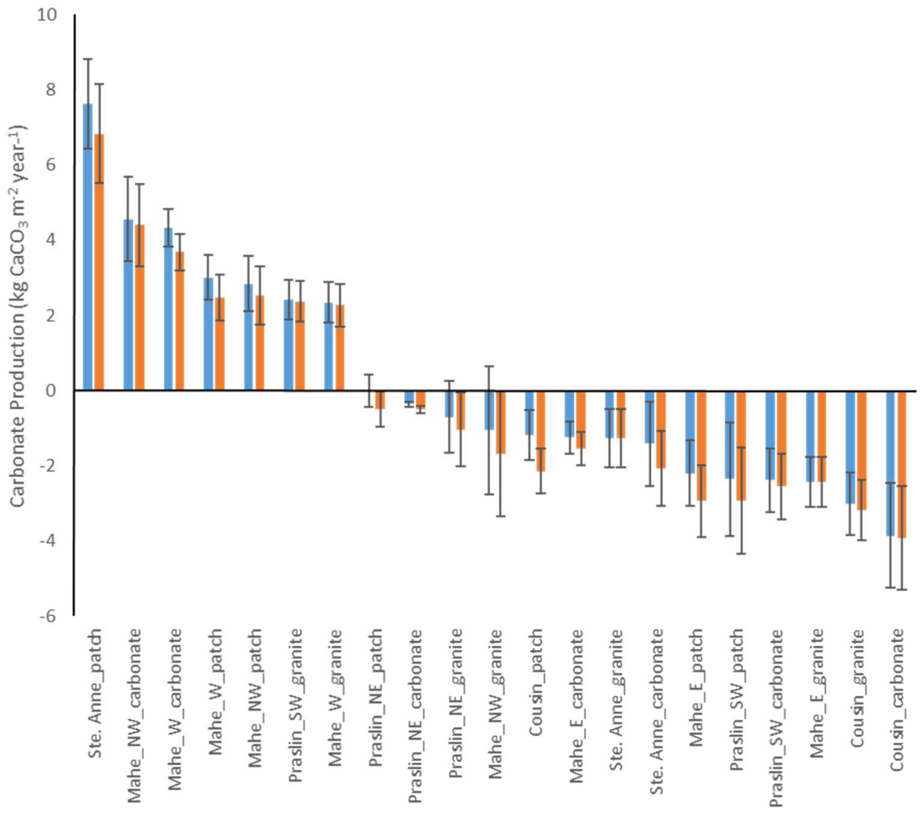

Figure S3| 2014 net carbonate balance across reefs excluding urchin erosion (blue) and including urchin erosion (red).

3. Multiple-regression analysis of carbonate production versus coral cover estimates and sensitivity analysis

Table S4| Multiple regression results for carbonate production relative to estimated cover of coral morphologies. Asterisks indicate significance level: * < 0.05; ** < 0.01; *** < 0.001.

Estimates	Model	R^2	AICc	Coefficients				
				Total Coral	Branching Coral	Encrusting Coral	Massive Coral	Table Coral
Mean	Total cover	0.87	229.1	0.053***	-	-	-	-
	Morphological cover	0.89	213.4	-	0.042***	0.048***	0.096***	0.107***
Lower 95% CI	Total	0.82	197.6	0.040***	-	-	-	-
	Morphological cover	0.85	175.8	-	0.030***	0.037***	0.088***	0.048*
Upper 95% CI	Total	0.88	287.1	0.065***	-	-	-	-
	Morphological cover	0.89	272.3	-	0.052***	0.059***	0.109***	0.155***

4. Sensitivity Analysis of Coral Growth Rates

We further calculated the 95% confidence intervals of these mean rates and calculated carbonate production based on these values as well. We then conducted two analyses. First, we repeated the multiple linear regression prediction, and linear mixed effects models for both carbonate balance and production with both the upper and lower 95% confidence interval values. Second, we produced a distribution of the mixed effect model coefficients by randomly sampling 9,999 times from the mean, upper and lower 95% CI hindcasted carbonate balance and production estimates and re-running the models. We then produced histograms of the distribution of co-efficients. No substantial differences were found for overall patterns of carbonate budget state between or within years using either 95% confidence intervals or random resampling, except that mean carbonate budget for all sites in 2011 was potentially not substantially different from 2005 or 2008.

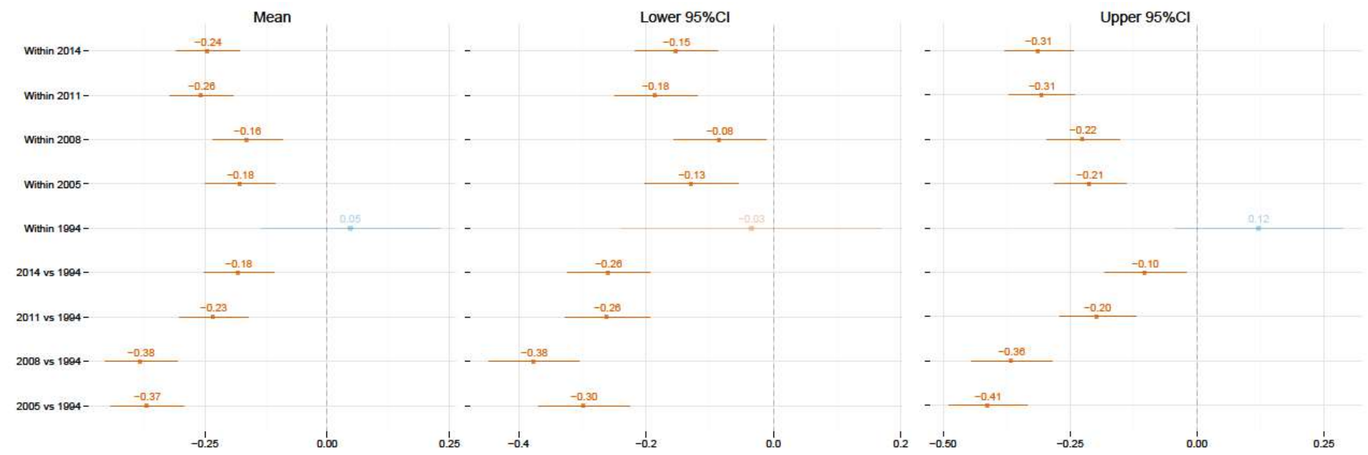

Figure S4| Forrest plots of standardised co-efficients and 95% confidence intervals for linear mixed effects models using mean, lower and upper 95% confidence intervals for genera specific growth rates for within year differences, and between each year and 1994. If confidence intervals overlap zero, it is indicative of there being no significant difference between groups.

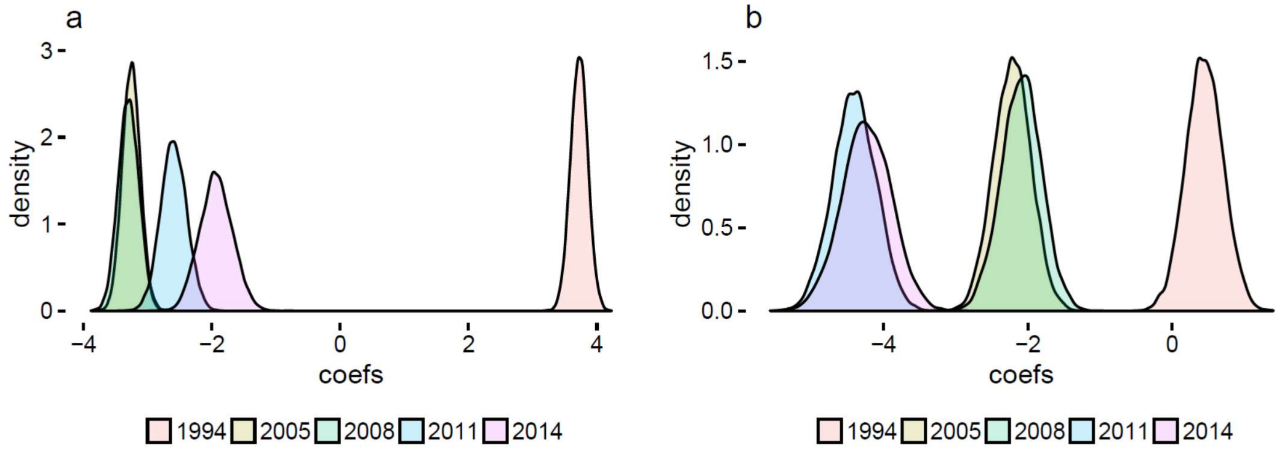

Figure S5| Distribution of coefficients (non-standardised) from bootstrapped resampling of estimates of carbonate balance based on mean, and upper and lower 95% confidence intervals: a) between years; and b) within years.

5. Boosted regression trees

Table S5| Predictor variables used in the boosted regression tree analysis

Variable	Description	Range (mean values at reef scale)		Justification
		1995	2005	
Scraper biomass	Scraper biomass in kg hectare^{-1}	16.9 – 170.9	17.4 – 296.1	Scraping parrotfishes scrape the surface of the reef, removing small amounts of reef substrate with each bite. They also play a role in preventing macroalgal proliferation [66,67].
Excavator biomass	Excavator biomass in kg hectare^{-1}	27.6 – 107.2	15.8 – 220.9	Excavating parrotfishes remove a portion of the reef substrate with each bite, and are often the primary bioeroders on coral reefs. Their impact scales exponentially with size, and therefore biomass is likely to be more closely associated with erosion than abundance [65,66].
Browser biomass	Browser biomass in kg hectare^{-1}	0 – 75.1	0 – 137.1	Browsing parrotfish crop vertical algae without removing substrate, and therefore can be important in maintaining space for coral colonisation.
Massive coral cover	Cover of corals with massive morphology in %	1.3 – 39.6	0 – 16.5	Massive corals are slow growing but primarily have life-history strategies that are more resistant to disturbance. They can also provide a degree of habitat complexity [52,53].
Branching coral cover	Cover of corals with branching morphology in %	2.8 – 48.4	0 – 6.6	Branching corals tend to be fast growing and can be the main source of carbonate production on healthy reefs. They also provide considerable habitat complexity and niche space for diverse organisms. However, they are more susceptible to disturbance (particularly *Acropora* sp.) and often show the highest mortality caused by bleaching [68,70,71].
Encrusting coral cover	Cover of corals with encrusting morphology in %	N/A	0 – 8.2	Encrusting corals are generally resilient to disturbance, and may expand post-disturbance. However, they have low carbonate production rates and provide little to no habitat complexity [68,72].
Depth	Depth in metres	2.9 – 10.0	2.9 – 10.0	Light intensity and temperature, greater in shallow water, are critical for both coral and macroalgae growth. Bioerosion by endolithic organisms can also vary with depth [73,74].
Wave exposure	Wave energy in J m^{-3}	0.04 – 0.57	0.04 – 0.57	Wave exposure influences coral distribution patterns, growth forms, colony sizes and persistence that will likely affect carbonate budgets [75,76].
Structural complexity	Topographic complexity of the substrate from scaled from 0 to 5	2.56 – 3.69	1.63 – 3.44	The structural complexity of a reef provides niche space for settlement and survival of a diverse range of organisms. It is also a broad measure of the complexity of coral morphologies, which can influence carbonate production rates [77].
Macroalgal cover	Presence/percent cover of fleshy macroalgae	Present/ Absent	0 - 47	Macroalgae cover often expands when new space is open to colonisation and can outcompete corals, preventing settlement, reducing growth rates and increasing mortality [78].
Habitat	Patch, carbonate or granitic reef	N/A	N/A	Three habitat types were surveyed in Seychelles, including carbonate fringing reefs, granitic rocky reefs, and patch reef habitats. These habitats differ in stability for recruitment of sessile larvae,

				susceptibility to erosion and levels of isolation [79,80].
Reserve status	No-take reserve or open for fishing	N/A	N/A	No-take marine reserves are expected to reduce fishing, increasing the abundance and biomass of parrotfishes, which are important bioeroders. They may also enhance other ecosystem processes and promote coral recovery [80-82].

5.1 Boosted Regression Tree Methods

Pairwise relationships between all variables for data from both 1994 and 2005 were assessed using Spearman rank correlation. There were significant correlations > 0.7 between all parrotfish biomass and scraper biomass for both 1994 and 2005, and between all parrotfish biomass and excavator biomass in 2005. There were also correlations > 0.7 between total coral cover and branching coral cover in 1994, and between total coral and the cover of branching, encrusting and massive coral in 2005 (Figure S6). We therefore ran BRT analyses for both 1994 and 2005 starting conditions for both combined coral and parrotfish variables, and for the sub-categorised variables.

Cross-validation (CV) deviance and standard error (se) were used to assess model performance (where lower values indicate a better model). Model optimisation was achieved by adjusting three parameters: tree complexity (tc – the number of nodes in a tree); learning rate (lr – establishes the contribution of each tree to the model); and bag-fraction (bf – specifies the proportion of data to be selected at each step). All possible combinations of tc (1, 2, 3, 4, 5), lr (0.01, 0.001, 0.005) and bf (0.5, 0.75) were run in a loop, and the combination with the lowest deviance was used to fit the final Boosted Regression Trees (BRT). Finally, we used the *gbm.simplify* routine in the *dismo* package [83] to perform a backward selection that drops variables contributing little (assessed using the average CV error to decide how many variables can be removed from the original model without affecting predictive performance) and therefore increases predictive efficiency.

Where we have given potential threshold values this is only in the cases where the shape of the partial dependence plots (which show the effect of a variable on the dependent variable after accounting for the average effect of the other variables) best matches the distribution of fitted values. The final BRT models for both pre-bleaching and post-collapse starting conditions excluded both habitat and reserve status variables in all cases.

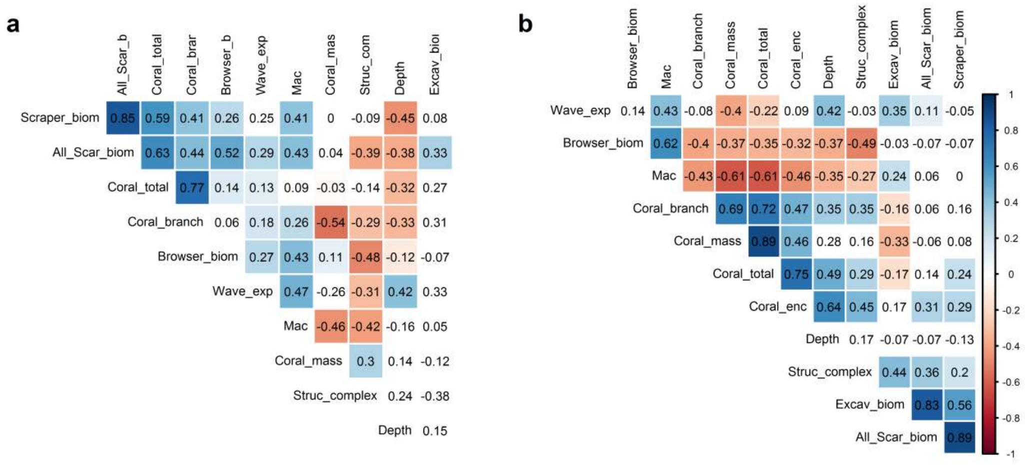

Figure S6| Correlation matrices for the ecological variables used in the Boosted Regression Tree models. (a) pre-bleaching (1994) starting conditions, and (b) post-collapse (2005) starting conditions.

Table S6| Influence of historical ecological conditions on current reef accretion regimes: optimal parameters and predictive performance of the final Boosted Regression Tree models. Cross-validation deviance (cv) and standard error (se) were used as measures of model performance (lower values indicate superior models)

Year	Categorisation	Number of predictor variables	Learning rate	Tree complexity	Bag-fraction	# of trees	Mean total deviance	CV	SE
1994	Ungrouped	9	0.01	1	0.75	1750	1.35	0.684	0.087
2005	Ungrouped	3	0.001	5	0.5	3050	1.358	0.735	0.077

6. Benthic cover results

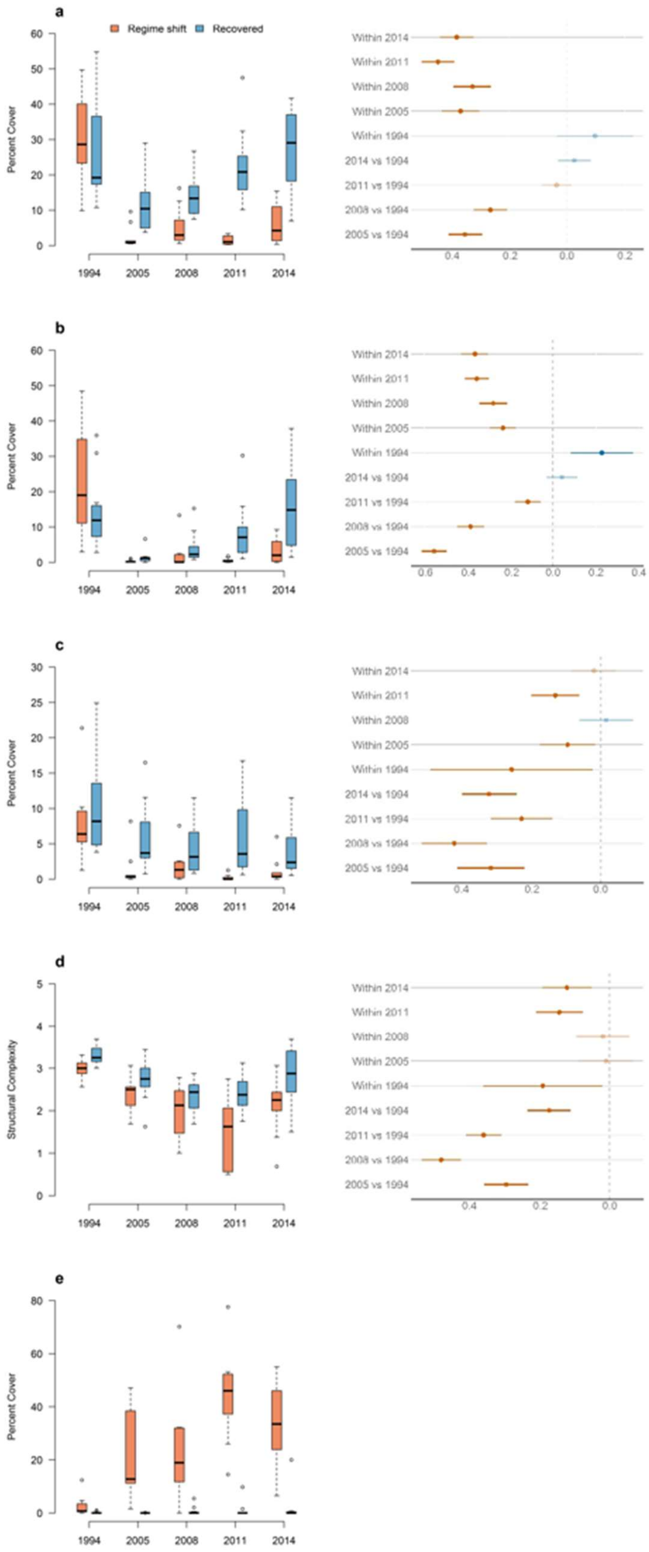

Figure S7| Benthic cover and forrest plots of standardised coefficients on Inner Seychelles reefs from 1994 – 2014. a, total coral; **b**, branching coral; **c**, massive coral; **d**, structural complexity and **e**, macroalgae, at 21 reefs in 1994, 2005, 2008, 2011 and 2014.

Table S7| Cover of massive coral and macroalgae, and budget and ecological state at each reef in 1994, 2005 and 2014.

Reef	Massive Coral (%)			Macroalgae (%)			Reef budget State 2014	Ecological State
	1994	2005	2014	1994	2005	2014		
Ste. Anne_patch	39.6	5.6	2.5	0.0	0.0	0.0	Positive	Recovered
Mahe_W_carbonate	24.9	10.6	8.9	0.0	0.0	0.8	Positive	Recovered
Mahe_NW_carbonate	7.7	5.1	4.9	0.0	0.0	0.0	Positive	Recovered
Mahe_W_patch	10.8	16.5	11.5	0.0	0.0	0.0	Positive	Recovered
Mahe_NW_patch	10.1	11.6	5.9	0.0	0.0	0.0	Positive	Recovered
Mahe_W_granite	8.0	3.8	0.9	0.0	0.0	0.0	Positive*	Recovered
Praslin_SW_granite	4.1	0.8	2.1	0.3	0.0	0.4	Positive*	Recovered
Praslin_NE_patch	8.4	3.4	5.9	0.0	0.0	0.0	Positive	Recovered
Praslin_NE_carbonate	21.4	0.5	0.3	0.0	11.2	46.0	Negative	Regime-shifted
Mahe_NW_granite	5.6	2.9	1.0	0.0	0.0	0.0	Negative†	Recovered
Praslin_NE_granite	3.9	1.0	0.5	0.0	0.0	0.1	Negative†	Recovered
Mahe_E_carbonate	5.3	8.2	6.0	2.0	12.8	33.5	Negative	Regime-shifted
Ste. Anne_carbonate	9.6	0.2	2.1	12.4	47.1	6.5	Negative	Regime-shifted
Ste. Anne_granite	3.8	3.6	2.0	1.0	0.1	20.0	Negative†	Recovered
Cousin_patch	6.8	0.1	0.3	0.0	35.0	23.9	Negative	Regime-shifted
Mahe_E_patch	16.4	3.1	2.3	0.6	0.1	0.4	Negative	Regime-shifted
Mahe_E_granite	6.4	2.5	0.6	0.7	1.6	40.4	Negative	Regime-shifted
Praslin_SW_carbonate	3.1	0.0	0.1	3.4	38.4	48.3	Negative	Regime-shifted
Praslin_SW_patch	5.6	0.4	0.4	4.7	12.0	11.9	Negative	Regime-shifted
Cousin_granite	10.2	0.5	0.9	0.6	3.6	29.8	Negative	Regime-shifted
Cousin_carbonate	1.3	0.3	0.0	0.4	41.3	55.0	Negative	Regime-shifted

*budget state was negative using growth rates from lower 95% CI.

†budget state was positive using growth rates from upper 95% CI.

Table S8| Reef abiotic and biotic characteristics 1994 – 2014

Year	Location	Habitat	Protection	Status	Wave exposure (kj m^{-3})	Depth	Structural complexity	Benthic cover (%)				Parrotfish biomass (kg hectare^{-1})			Carbonate production rate (kg $CaCO_3$ m^{-2} yr^{-1})	
								Branched coral	Encrusting coral	Massive coral	Macroalgae	Browsing	Scraping	Excavating	Carbonate production	Total Erosion
1994	Cousin	carbonate	Reserve	Regime shift	0.57	5.98	3.06	48.44	0.00	1.25	0.44	5.03	81.78	24.33	7.66	-1.63
1994	Cousin	granite	Reserve	Regime shift	0.13	5.61	3.00	12.50	0.00	10.19	0.56	10.20	82.42	20.06	4.86	-1.38
1994	Cousin	patch	Reserve	Regime shift	0.49	6.69	3.13	31.88	0.00	6.75	0.00	6.88	76.89	17.45	6.67	-1.80
1994	Mahe_E	carbonate	Fished	Regime shift	0.49	6.19	2.81	19.00	0.00	9.63	12.38	12.09	56.97	11.48	5.51	-2.48
1994	Mahe_E	granite	Fished	Regime shift	0.50	9.36	2.56	2.94	0.00	6.38	0.69	14.42	42.03	7.10	2.35	-1.45
1994	Mahe_E	patch	Fished	Recovery	0.50	8.61	3.56	2.81	0.00	16.38	0.56	4.45	61.81	17.44	4.92	-2.68
1994	Mahe_NW	carbonate	Reserve	Recovery	0.12	6.13	3.00	30.94	0.00	7.69	0.00	0.00	42.71	25.07	6.78	-2.27
1994	Mahe_NW	granite	Fished	Recovery	0.13	6.83	3.19	5.13	0.00	5.63	0.00	5.73	35.05	21.50	2.32	-2.05
1994	Mahe_NW	patch	Fished	Recovery	0.08	5.58	3.31	7.06	0.06	10.13	0.00	5.11	16.85	24.05	3.85	-1.41
1994	Mahe_W	carbonate	Fished	Recovery	0.04	5.89	3.25	9.31	0.00	24.94	0.00	0.00	42.01	23.49	8.25	-1.23
1994	Mahe_W	granite	Fished	Recovery	0.16	8.47	3.38	11.25	0.00	8.00	0.00	3.68	47.11	30.19	3.89	-1.83
1994	Mahe_W	patch	Fished	Recovery	0.22	6.50	3.13	7.50	0.00	10.75	0.00	15.16	48.27	26.52	4.08	-1.81
1994	Praslin_NE	carbonate	Fished	Regime shift	0.07	4.51	3.31	6.88	0.00	21.38	0.00	11.50	83.50	8.94	6.90	-1.80
1994	Praslin_NE	granite	Fished	Recovery	0.11	4.87	3.25	12.50	0.00	3.88	0.00	10.78	46.69	19.66	2.95	-1.58
1994	Praslin_NE	patch	Fished	Recovery	0.13	7.08	3.56	16.88	0.00	8.38	0.00	21.26	35.93	7.29	4.85	-1.63
1994	Praslin_SW	carbonate	Fished	Regime shift	0.35	6.26	2.88	39.00	0.00	3.13	3.44	0.00	54.77	12.86	6.75	-2.09
1994	Praslin_SW	granite	Fished	Recovery	0.18	7.14	3.69	13.38	0.00	4.06	0.25	6.22	56.11	19.78	3.14	-1.35
1994	Praslin_SW	patch	Fished	Regime shift	0.18	4.93	2.94	11.13	0.00	5.56	4.69	0.00	49.90	24.22	3.21	-1.79
1994	Ste. Anne	carbonate	Reserve	Regime shift	0.06	5.57	3.19	34.81	0.00	5.25	2.00	0.00	170.94	15.95	6.70	-1.55
1994	Ste. Anne	granite	Reserve	Recovery	0.49	7.11	3.13	35.94	0.00	3.81	1.00	12.28	76.76	14.09	6.48	-2.56
1994	Ste. Anne	patch	Reserve	Recovery	0.46	7.61	3.25	15.19	0.00	39.63	0.00	26.39	111.34	38.24	13.16	-2.23
2005	Cousin	carbonate	Reserve	Regime shift	0.57	4.45	2.50	0.00	0.31	0.31	41.31	13.85	136.48	22.34	0.11	-3.78
2005	Cousin	granite	Reserve	Regime shift	0.13	4.93	2.13	0.19	0.31	0.50	3.56	24.74	61.26	25.91	0.19	-0.57
2005	Cousin	patch	Reserve	Regime shift	0.49	6.19	2.75	0.06	0.31	0.13	34.97	21.59	44.31	22.60	0.08	-2.32
2005	Mahe_E	carbonate	Fished	Regime shift	0.49	6.15	2.50	0.19	0.75	0.19	47.09	12.56	32.61	18.02	0.17	-1.75
2005	Mahe_E	granite	Fished	Regime shift	0.50	9.38	2.00	1.06	2.63	2.50	1.56	1.55	45.84	26.88	1.16	-1.34

2005	Mahe_E	patch	Fished	Recovery	0.50	7.71	2.63	0.63	1.75	3.06	0.13	3.66	61.17	20.52	1.10	-2.76
2005	Mahe_NW	carbonate	Reserve	Recovery	0.12	5.54	2.94	0.38	3.88	5.06	0.00	3.06	66.33	37.31	1.80	-1.77
2005	Mahe_NW	granite	Fished	Recovery	0.13	7.33	2.31	0.00	6.31	2.94	0.00	0.00	147.06	24.91	1.39	-1.52
2005	Mahe_NW	patch	Fished	Recovery	0.08	6.21	2.88	0.50	1.88	11.56	0.00	10.23	17.39	10.12	3.42	-0.85
2005	Mahe_W	carbonate	Fished	Recovery	0.04	5.64	2.56	1.63	2.38	10.56	0.00	0.03	151.38	27.72	3.36	-1.17
2005	Mahe_W	granite	Fished	Recovery	0.16	10.04	3.44	1.44	7.13	3.75	0.00	6.66	106.78	31.13	1.90	-2.25
2005	Mahe_W	patch	Fished	Recovery	0.22	7.48	2.88	6.63	3.50	16.50	0.00	11.48	39.65	26.83	5.88	-0.86
2005	Praslin_NE	carbonate	Fished	Regime shift	0.07	4.24	1.69	0.00	0.00	0.50	11.19	0.00	33.10	32.44	0.14	-0.68
2005	Praslin_NE	granite	Fished	Recovery	0.11	5.23	3.44	1.25	1.50	1.00	0.00	15.12	70.83	42.76	0.60	-2.50
2005	Praslin_NE	patch	Fished	Recovery	0.13	8.48	2.56	0.13	0.75	3.44	0.00	11.14	34.54	20.09	1.03	-1.44
2005	Praslin_SW	carbonate	Fished	Regime shift	0.35	6.63	2.56	0.00	0.44	0.00	38.44	9.57	68.77	37.62	0.08	-1.68
2005	Praslin_SW	granite	Fished	Recovery	0.18	6.64	2.56	1.13	1.63	0.75	0.00	8.42	58.57	41.92	0.62	-1.22
2005	Praslin_SW	patch	Fished	Regime shift	0.18	5.06	3.06	0.19	0.19	0.38	12.00	0.00	64.44	23.14	0.15	-2.82
2005	Ste. Anne	carbonate	Reserve	Regime shift	0.06	2.86	2.31	0.69	0.25	8.19	12.75	0.00	120.86	20.30	2.37	-1.31
2005	Ste. Anne	granite	Reserve	Recovery	0.49	7.18	3.06	1.38	8.19	3.63	0.06	9.03	296.91	51.61	2.03	-2.98
2005	Ste. Anne	patch	Reserve	Recovery	0.46	6.76	1.63	1.31	0.25	5.56	0.00	15.63	57.83	35.68	1.75	-0.48
2008	Cousin	carbonate	Reserve	Regime shift	0.57	3.76	2.47	0.06	0.81	0.19	32.00	2.12	97.04	14.67	0.15	-2.00
2008	Cousin	granite	Reserve	Regime shift	0.13	5.50	1.47	0.00	0.44	2.56	24.63	10.75	75.76	19.33	0.74	-2.49
2008	Cousin	patch	Reserve	Regime shift	0.49	5.83	2.34	0.00	0.44	0.06	32.19	2.77	108.34	16.58	0.06	-4.28
2008	Mahe_E	carbonate	Fished	Regime shift	0.49	5.84	2.78	13.31	1.13	1.31	2.44	0.00	49.03	29.83	2.58	-2.81
2008	Mahe_E	granite	Fished	Regime shift	0.50	10.24	2.09	2.19	2.31	2.44	0.00	5.70	48.13	48.15	1.21	-1.97
2008	Mahe_E	patch	Fished	Recovery	0.50	7.73	2.53	1.69	2.75	3.44	2.06	3.86	117.36	26.60	1.49	-2.34
2008	Mahe_NW	carbonate	Reserve	Recovery	0.12	Not collected	2.53	2.13	1.50	3.81	0.00	0.44	88.13	16.19	1.50	-1.50
2008	Mahe_NW	granite	Fished	Recovery	0.13	Not collected	2.06	1.25	5.88	1.31	0.00	17.38	169.83	15.85	1.09	-4.00
2008	Mahe_NW	patch	Fished	Recovery	0.08	Not collected	2.81	1.63	0.75	8.56	0.00	25.57	33.37	41.56	2.66	-1.08
2008	Mahe_W	carbonate	Fished	Recovery	0.04	Not collected	2.41	2.38	0.75	11.50	0.00	21.85	70.90	17.65	3.60	-1.50
2008	Mahe_W	granite	Fished	Recovery	0.16	Not collected	2.41	1.19	16.38	0.88	0.00	6.16	81.74	16.71	2.00	-1.72
2008	Mahe_W	patch	Fished	Recovery	0.22	Not collected	2.06	5.75	0.31	8.50	0.00	3.21	42.84	36.93	3.35	-1.33
2008	Praslin_NE	carbonate	Fished	Regime shift	0.07	4.12	1.16	0.06	0.50	1.81	18.94	0.00	14.38	17.63	0.55	-0.87

2008	Praslin_NE	granite	Fished	Recovery	0.11	4.83	2.88	15.25	3.31	0.81	0.00	14.41	64.15	31.56	4.43	-1.37
2008	Praslin_NE	patch	Fished	Recovery	0.13	9.02	1.69	0.81	1.31	4.75	0.00	10.23	69.38	33.31	1.55	-1.21
2008	Praslin_SW	carbonate	Fished	Regime shift	0.35	7.73	1.00	0.06	0.13	0.00	70.19	0.00	9.04	34.66	0.06	-1.20
2008	Praslin_SW	granite	Fished	Recovery	0.18	6.83	2.47	3.19	3.81	1.25	0.06	8.70	216.20	29.16	3.38	-4.50
2008	Praslin_SW	patch	Fished	Regime shift	0.18	4.80	2.66	0.56	0.81	1.06	11.69	0.00	191.52	21.65	0.49	-2.79
2008	Ste. Anne	carbonate	Reserve	Regime shift	0.06	Not collected	2.13	2.50	1.88	7.56	18.88	4.49	31.88	7.61	2.68	-1.37
2008	Ste. Anne	granite	Reserve	Recovery	0.49	6.81	2.69	2.50	9.19	1.56	0.50	8.38	199.69	13.02	1.76	-2.91
2008	Ste. Anne	patch	Reserve	Recovery	0.46	Not collected	2.06	8.94	1.00	2.88	5.44	0.00	40.77	34.37	2.30	-1.91
2011	Cousin	carbonate	Reserve	Regime shift	0.57	Not collected	2.50	0.00	0.38	0.00	49.25	4.64	74.22	27.07	0.03	-4.53
2011	Cousin	granite	Reserve	Regime shift	0.13	Not collected	1.63	0.63	0.88	1.25	37.25	9.17	85.12	28.72	0.52	-3.77
2011	Cousin	patch	Reserve	Regime shift	0.49	Not collected	2.75	1.75	1.00	0.00	26.00	0.00	87.74	17.85	0.36	-5.93
2011	Mahe_E	carbonate	Fished	Regime shift	0.49	Not collected	1.44	0.50	1.38	0.00	52.25	7.07	36.86	11.19	0.20	-3.00
2011	Mahe_E	granite	Fished	Regime shift	0.50	Not collected	1.94	0.38	0.63	0.00	46.00	0.35	75.94	21.56	0.11	-2.84
2011	Mahe_E	patch	Fished	Recovery	0.50	Not collected	2.13	1.00	6.13	5.00	0.13	2.21	261.75	4.15	2.12	-4.79
2011	Mahe_NW	carbonate	Reserve	Recovery	0.12	Not collected	2.13	15.88	0.75	9.63	0.00	15.18	246.33	41.56	5.25	-3.21
2011	Mahe_NW	granite	Fished	Recovery	0.13	Not collected	2.63	2.88	14.38	1.50	0.00	13.46	142.23	61.49	2.24	-1.87
2011	Mahe_NW	patch	Fished	Recovery	0.08	Not collected	2.38	9.00	1.63	10.00	0.00	20.24	40.66	50.18	4.43	-1.79
2011	Mahe_W	carbonate	Fished	Recovery	0.04	Not collected	2.38	6.88	0.88	16.13	0.00	24.48	279.32	18.09	5.54	-3.61
2011	Mahe_W	granite	Fished	Recovery	0.16	Not collected	2.75	7.25	20.13	2.75	0.00	28.61	122.26	36.77	4.35	-2.87
2011	Mahe_W	patch	Fished	Recovery	0.22	Not collected	2.13	30.25	0.50	16.75	0.00	12.15	65.42	24.71	9.21	-2.55
2011	Praslin_NE	carbonate	Fished	Regime shift	0.07	Not collected	0.50	0.00	0.50	0.50	53.13	0.00	3.47	33.90	0.18	-0.60
2011	Praslin_NE	granite	Fished	Recovery	0.11	Not collected	3.13	6.75	14.38	0.63	0.13	18.01	210.33	42.24	3.09	-2.68
2011	Praslin_NE	patch	Fished	Recovery	0.13	Not collected	1.75	2.25	3.50	4.38	0.00	12.54	47.57	14.92	1.86	-1.26
2011	Praslin_SW	carbonate	Fished	Regime shift	0.35	Not collected	0.56	0.13	0.25	0.00	77.63	1.35	63.49	6.81	0.04	-1.16
2011	Praslin_SW	granite	Fished	Recovery	0.18	Not collected	2.50	8.25	4.38	2.38	0.00	16.06	81.67	8.57	3.02	-2.82
2011	Praslin_SW	patch	Fished	Regime shift	0.18	Not collected	2.06	1.38	1.50	0.25	14.50	19.71	216.61	35.01	0.49	-3.89

2011	Ste. Anne	carbonate	Reserve	Regime shift	0.06	Not collected	0.56	0.13	0.13	0.00	40.00	6.96	9.35	20.97	0.03	-1.77
2011	Ste. Anne	granite	Reserve	Recovery	0.49	Not collected	3.00	2.75	15.38	1.25	1.50	7.48	226.65	32.75	2.46	-5.54
2011	Ste. Anne	patch	Reserve	Recovery	0.46	Not collected	1.94	11.00	0.38	2.00	9.75	0.00	94.95	28.27	2.46	-1.57
2014	Cousin	carbonate	Reserve	Regime shift	0.57	4.45	2.38	0.13	0.38	0.00	55.00	0.00	4.16	22.85	0.05	-3.90
2014	Cousin	granite	Reserve	Regime shift	0.13	4.93	2.44	0.25	0.25	0.88	29.75	0.00	68.78	18.01	0.30	-3.21
2014	Cousin	patch	Reserve	Regime shift	0.49	6.19	3.06	8.88	1.88	0.25	23.88	0.00	138.34	19.17	1.58	-3.13
2014	Mahe_E	carbonate	Fished	Regime shift	0.49	6.15	2.00	3.00	1.38	2.13	6.50	0.00	52.33	45.90	1.27	-2.40
2014	Mahe_E	granite	Fished	Regime shift	0.50	9.38	1.38	0.75	0.25	0.63	40.38	0.44	44.61	12.39	0.38	-2.79
2014	Mahe_E	patch	Fished	Recovery	0.50	7.71	2.19	1.44	3.00	2.25	0.38	9.48	139.83	34.03	1.18	-3.12
2014	Mahe_NW	carbonate	Reserve	Recovery	0.12	5.54	2.31	32.00	0.88	4.88	0.00	5.89	105.03	25.25	6.36	-2.84
2014	Mahe_NW	granite	Fished	Recovery	0.13	7.33	3.69	6.50	16.13	1.00	0.00	19.53	97.55	39.99	2.74	-3.61
2014	Mahe_NW	patch	Fished	Recovery	0.08	6.21	2.56	19.13	0.50	5.88	0.00	21.03	52.82	24.30	4.58	-1.98
2014	Mahe_W	carbonate	Fished	Recovery	0.04	5.64	2.88	27.13	2.13	8.88	0.00	54.76	385.62	2.41	6.73	-2.61
2014	Mahe_W	granite	Fished	Recovery	0.16	10.04	3.38	10.50	19.88	0.88	0.00	0.00	112.20	0.00	5.02	-3.14
2014	Mahe_W	patch	Fished	Recovery	0.22	7.48	2.88	19.75	0.75	11.50	0.00	0.00	52.13	0.00	6.35	-2.93
2014	Praslin_NE	carbonate	Fished	Regime shift	0.07	4.24	0.69	0.00	0.13	0.25	46.00	0.79	8.28	40.37	0.08	-0.41
2014	Praslin_NE	granite	Fished	Recovery	0.11	5.23	3.25	10.50	10.38	0.50	0.13	0.00	115.22	0.00	3.04	-4.10
2014	Praslin_NE	patch	Fished	Recovery	0.13	8.48	1.50	2.00	1.50	5.88	0.00	0.00	49.02	0.00	2.05	-1.38
2014	Praslin_SW	carbonate	Fished	Regime shift	0.35	6.63	2.25	2.00	1.88	0.13	48.25	12.89	70.06	27.82	0.58	-3.03
2014	Praslin_SW	granite	Fished	Recovery	0.18	6.64	3.63	19.50	7.63	2.13	0.38	0.00	76.47	0.00	5.20	-3.69
2014	Praslin_SW	patch	Fished	Regime shift	0.18	5.06	2.63	5.88	2.75	0.38	11.88	17.74	159.38	23.93	1.93	-4.72
2014	Ste. Anne	carbonate	Reserve	Regime shift	0.06	2.86	2.06	9.25	0.13	6.00	33.50	19.19	90.49	43.10	3.06	-4.17
2014	Ste. Anne	granite	Reserve	Recovery	0.49	7.18	3.44	3.13	5.13	2.00	20.00	251.06	0.00	0.00	2.53	-3.97
2014	Ste. Anne	patch	Reserve	Recovery	0.46	6.76	2.75	40.00	0.75	2.50	0.75	460.08	0.00	0.00	7.09	-1.28

References

1. Perry, C. T., Spencer, T. & Kench, P. S. 2008 Carbonate budgets and reef production states: a geomorphic perspective on the ecological phase-shift concept. *Coral Reefs* **27**, 853–866. (doi:10.1007/s00338-008-0418-z)
2. Perry, C. T., Murphy, G. N., Graham, N. A. J., Wilson, S. K., Januchowski-Hartley, F. A. & East, H. K. 2015 Remote coral reefs can sustain high growth potential and may match future sea-level trends. Sci. Rep. 5, 18289. (doi:10.1038/srep18289)
3. Harriott, V. J. (1999). Coral growth in subtropical eastern Australia. Coral Reefs, 18(3), 281–291. doi:10.1007/s003380050195
4. Browne, N. K. (2012). Spatial and temporal variations in coral growth on an inshore turbid reef subjected to multiple disturbances. *Marine Environmental Research*, 77, 71–83. doi:10.1016/j.marenvres.2012.02.005
5. Bucher, D. J., Harriott, V. J., & Roberts, L. G. (1998). Skeletal micro-density, porosity and bulk density of acroporid corals. Journal of Experimental Marine Biology and Ecology, 228(1), 117–136. doi:10.1016/S0022-0981(98)00020-4
6. Charuchinda M, Hylleberg J (1984) Skeletal extension of Acropora formosa at a fringing reef in the Andaman Sea. Coral Reefs 17: 215–219. Available: http://link.springer.com/article/10.1007/BF00288257. Accessed 14 May 2014.
7. Crossland C (1981) Seasonal growth of Acropora cf. formosa and Pocillopora damicornis on a high latitude reef (Houtman Abrolhos, Western Australia). Proc 4th int coral Reef Symp 1: 663–667. Available: https://publications.csiro.au/rpr/pub?list=BRO&pid=procite:db4a6536-a1af-4919-b8d1-2357707ddd4d. Accessed 30 May 2014.
8. Morgan KM & Kench PS 2012. Skeletal extension and calcification of reef-building corals in the central Indian Ocean. Marine Environmental Research. 81, 78-82.
9. Neudecker S (1981) Growth and survival of scleractinian corals exposed to thermal effluents at Guam. Available: http://agris.fao.org/agris-search/search.do?recordID=XB8311021. Accessed 9 December 2014.
10. Oliver J, Chalker B, Dunlap W (1983) Bathymetric adaptations of reef-building corals at davies reef, great barrier reef, Australia. I. Long-term growth responses of Acropora formosa(Dana 1846). J Exp Mar Biol … 73: 11–35. Available: http://www.sciencedirect.com/science/article/pii/0022098183900035. Accessed 19 May 2014.
11. Roche, R. C., Abel, R. a., Johnson, K. G., & Perry, C. T. (2010). Quantification of porosity in Acropora pulchra (Brook 1891) using X-ray micro-computed tomography techniques. Journal of Experimental Marine Biology and Ecology, 396(1), 1–9. doi:10.1016/j.jembe.2010.10.006
12. Yap H, Gomez E (1985) Growth of Acropora pulchra. Mar Biol 87: 203–209. Available: http://link.springer.com/article/10.1007/BF00539430. Accessed 9 December 2014.
13. Marsh L (1993) The occurrence and growth of Acropora in extra-tropical waters off Perth, Western Australia. Proc 7th Int Coral Reef … 2: 1233–1238. Available: http://scholar.google.com/scholar?hl=en&btnG=Search&q=intitle:The+occurrence+and+growth+of+Acropora+in+extra-tropical+waters+off+Perth,+Western+Australia#0. Accessed 30 May 2014.
14. Shi, Q., Zhao, M., Zhang, Q., Yu, K., Chen, T., Li, S., & Wang, H. (2008). Estimate of carbonate production by scleractinian corals at Luhuitou fringing reef, Sanya, China. Chinese Science Bulletin, 54(4), 696–705. doi:10.1007/s11434-008-0533-9
15. Plucer-Rosario G, Randall R (1987) Preservation of rare coral species by transplantation and examination of their recruitment and growth. Bull Mar Sci 41: 585–593. Available: http://www.ingentaconnect.com/content/umrsmas/bullmar/1987/00000041/00000002/art00043. Accessed 9 December 2014.
16. Jokiel, P., & Tyler, W. (1992). Distribution of stony corals in Johnston Atoll lagoon. In Proc. Seventh Int. Coral Reef Symp (Vol. 2)
17. Brown, B., Sya'Rani, L., & Tissier, M. Le. (1985). Skeletal form and growth in Acropora aspera(Dana) from the Pulau Seribu, Indonesia. Journal of Experimental Marine Biology and Ecology, 86, 139–150. Retrieved from http://www.sciencedirect.com/science/article/pii/0022098185900279
18. Ma TYH (1958) The relation of growth rate of reef corals to surface temperature of sea water as a basis for study of causes of disastrophisms instigating evolution of life. World Book Co, LTD, Taipei, Taiwan. IN Jokiel and Tyler (1992)
19. Stimson J (1996) Wave like outward growth of some table- and plate- forming corals, and a hypothetical mechanism. Bulletin of Marine Sicence 58: 301–313.
20. Bessat, F., & Buigues, D. (2001). Two centuries of variation in coral growth in a massive Porites colony from Moorea (French Polynesia): a response of ocean-atmosphere variability from south. Palaeogeography, Palaeoclimatology, …, 175, 381–392. Retrieved from http://www.sciencedirect.com/science/article/pii/S0031018201003819
21. Buddemeier R, Maragos J, Knutson D (1974) Radiographic studies of reef coral exoskeletons: rates and patterns of coral growth. J Exp … 14. Available: http://www.sciencedirect.com/science/article/pii/0022098174900240. Accessed 9 December 2014.
22. Cabral-Tena, R., Reyes-Bonilla, H., Lluch-Cota, S., Paz-García, D., Calderón-Aguilera, L., Norzagaray-López, O., & Balart, E. (2013). Different calcification rates in males and females of the coral Porites panamensis in the Gulf of California. Marine Ecology Progress Series, 476, 1–8. doi:10.3354/meps10269
23. Cantin, N. E., & Lough, J. M. (2014). Surviving coral bleaching events: porites growth anomalies on the Great Barrier Reef. PloS One, 9(2), e88720. doi:10.1371/journal.pone.0088720

24. Chen, T., Li, S., Yu, K., Zheng, Z., Wang, L., & Chen, T. (2013). Increasing temperature anomalies reduce coral growth in the Weizhou Island, northern South China Sea. Estuarine, Coastal and Shelf Science, 130, 121–126. doi:10.1016/j.ecss.2013.05.009
25. Cooper, T. F., De'Ath, G., Fabricius, K. E., & Lough, J. M. (2008). Declining coral calcification in massive Porites in two nearshore regions of the northern Great Barrier Reef. Global Change Biology, 14(3), 529–538. doi:10.1111/j.1365-2486.2007.01520.x
26. De'ath, G., Lough, J., & Fabricius, K. (2009). Declining coral calcification on the Great Barrier Reef. Science, (November), 116–120. Retrieved from http://www.sciencemag.org/content/323/5910/116.short
27. Grottoli A. G (1999) Variability of stable isotopes and maximum linear extension in reef-coral skeletons at Kaneohe Bay, Hawaii. Mar Biol 135: 437–449. Available: http://link.springer.com/10.1007/s002270050644.
28. Grove CA, Nagtegaal R, Zinke J, Sceufen T, Koster B, Kasper S, McCulloch MT, van den Bergh G, Brummer G Jan A 2010. River runoff reconstructions from novel spectral luminescence scanning of massive coral skeletons. Coral Reefs. 29, 579-591.
29. Guzmán H, Cortés J (1989) Growth rates of eight species of scleractinian corals in the eastern Pacific (Costa Rica). Bull Mar Sci 44: 1186–1194. Available: http://www.ingentaconnect.com/content/umrsmas/bullmar/1989/00000044/00000003/art00010. Accessed 9 December 2014.
30. Highsmith R (1979) Coral growth rates and environmental control of density banding. J Exp Mar Bio Ecol 37: 105–125. Available: http://www.sciencedirect.com/science/article/pii/0022098179900893. Accessed 9 December 2014.
31. Klein R, Loya Y (1991) Skeletal growth and density patterns of two Porites corals from the Gulf of Eilat, Red Sea. Mar Ecol Prog Ser Oldend 77: 253–259. Available: http://www.researchgate.net/publication/250215112_
32. Lough, J., & Barnes, D. (1992). Comparisons of skeletal density variations in Porites from the central Great Barrier Reef. Journal of Experimental Marine Biology and Ecology, 155(1), 1–25. doi:10.1016/0022-0981(92)90024-5
33. Merschel G 2012. Reconstructing Past Climate Signals of northern Mozambique using Giant Corals. Masters thesis. VU University, Amseterdam.
34. Smith, L. W., Barshis, D., & Birkeland, C. (2007). Phenotypic plasticity for skeletal growth, density and calcification of Porites lobata in response to habitat type. Coral Reefs, 26(3), 559–567. doi:10.1007/s00338-007-0216-z
35. Watanabe T, Gagan MK, Correge T, Scott-Gagan H, Cowley J, Hantoro WS. 2003. Oxygen isotope systematics in Diploastrea heliopora: New coral archive of tropical paleoclimate. Geochimica et Cosmochimica Acta 67: 1349-1358.
36. Roberts LG, Harriott VJ (2003) Can environmental records be extracted from coral skeletons from Moreton Bay, Australia, a subtropical, turbid environment? Coral Reefs 22: 517–522. Available: http://link.springer.com/10.1007/s00338-003-0337-y. Accessed 24 November 2014.
37. Ma, 1958 in: Jokiel, P., & Tyler, W. (1992). Distribution of stony corals in Johnston Atoll lagoon. In Proc. Seventh Int. Coral Reef Symp (Vol. 2)
38. Babcock R (1988) Age-structure, survivorship and fecundity in populations of massive corals. Proc 6th int coral Reef Symp. Available: http://scholar.google.com/scholar?hl=en&btnG=Search&q=intitle:Age-structure,+survivorship+and+fecundity+in+populations+of+massive+corals#0. Accessed 9 December 2014.
39. Anthony, K. R. N., Connolly, S. R., & Willis, B. L. (2002). Comparative analysis of energy allocation to tissue and skeletal growth in corals. Limnology and Oceanography, 47(5), 1417–1429. doi:10.4319/lo.2002.47.5.1417
40. Weber J, White E (1974) Activation energy for skeletal aragonite deposited by the hermatypic coral Platygyra spp. Mar Biol 359: 353–359. Available: http://link.springer.com/article/10.1007/BF00391518. Accessed 9 December 2014.
41. Cox E (1986) The effects of a selective corallivore on growth rates and competition for space between two species of Hawaiian corals. J Exp Mar Bio Ecol 101: 161–174. Available: http://www.sciencedirect.com/science/article/pii/002209818690047X. Accessed 10 December 2014.
42. Manzello, D. P. (2010). Coral growth with thermal stress and ocean acidification: lessons from the eastern tropical Pacific. Coral Reefs, 29(3), 749–758. doi:10.1007/s00338-010-0623-4
43. Wellington G (1982) An experimental analysis of the effects of light and zooplankton on coral zonation. Oecologia: 311–320. Available: http://link.springer.com/article/10.1007/BF00367953. Accessed 9 December 2014.
44. Crossland C (1981) Seasonal growth of Acropora cf. formosa and Pocillopora damicornis on a high latitude reef (Houtman Abrolhos, Western Australia). Proc 4th int coral Reef Symp 1: 663–667. Available: https://publications.csiro.au/rpr/pub?list=BRO&pid=procite:db4a6536-a1af-4919-b8d1-2357707ddd4d. Accessed 30 May 2014.
45. Glynn P, Wellington G, Birkeland C (1979) Coral reef growth in the Galapagos: limitation by sea urchins. Science (80-): 8–10. Available: http://www.sciencemag.org/content/203/4375/47.short. Accessed 9 December 2014.
46. Neudecker S (1981) Growth and survival of scleractinian corals exposed to thermal effluents at Guam. Available: http://agris.fao.org/agris-search/search.do?recordID=XB8311021. Accessed 9 December 2014.
47. Ward S (1995) The effect of damage on the growth, reproduction and storage of lipids in the scleractinian coral Pocillopora damicornis (Linnaeus). J Exp Mar Bio Ecol 187: 193–206. Available: http://linkinghub.elsevier.com/retrieve/pii/002209819400180L.
48. Davies, P. (1989). Short-term growth measurements of corals using an accurate buoyant weighing technique. Marine Biology, 395, 389–395. Retrieved from http://link.springer.com/article/10.1007/BF00428135fkl

49. Chazottes, V., Le Campion-Almusard, T., Peyrot-Clausade, M. & Cuet, P. 2002 The effects of eutrophication-related alterations to coral reef communities on agents and rates of bioerosion (Reunion Island, Indian Ocean). *Coral reefs* **21**, 375–390. (doi:10.1007/s00338-002-0259-0)
50. Chazottes, V., Campion-Alsumard, T. L. & Peyrot-Clausade, M. 1995 Bioerosion rates on coral reefs: interactions between macroborers, microborers and grazers (Moorea, French Polynesia). *Palaeogeogr. Palaeoclimatol. Palaeoecol.* **113**, 189–198. (doi:10.1016/0031-0182(95)00043-L)
51. Tribollet, A. & Golubic, S. 2005 Cross-shelf differences in the pattern and pace of bioerosion of experimental carbonate substrates exposed for 3 years on the northern Great Barrier Reef, Australia. *Coral Reefs* **24**, 422–434. (doi:10.1007/s00338-005-0003-7)
52. Carreiro-Silva, M. & McClanahan, T. 2012 Macrobioerosion of dead branching Porites, 4 and 6 years after coral mass mortality. Mar. Ecol. Prog. Ser. 458, 103–122. (doi:10.3354/meps09726)
53. Polunin, N. V. C. & Roberts, C. M. 1993 Greater biomass and value of target coral-reef fishes in two small Caribbean marine reserves. *Mar. Ecol. Prog. Ser.* **100**, 167–176. (doi:10.3354/meps100167)
54. Conand, C., Chabanet, P., Cuet, P., & Letourneur, Y. 1987 The carbonate budget of a fringing reef in La Reunion Island (Indian Ocean): sea urchin and fish bioerosion and net calcification. In Proceedings of the 8th International Coral Reef Symposium (eds. Lessios, H. A., & Macintyre, I. G., Vol. 1, Smithsonian Tropical Research Institute, Panama pp. 953-958.
55. Russo, A.R. 1980 Bioerosion by two rock boring echinoids (*Echinometra mathaei* and *Echinostrephus aciculatus*) on Enewetak Atoll, Marshall Islands. J. Mar. Res. 38, 99-110.
56. Downing, N. & El-Zahr, C. 1987 Gut evacuation and filling rates in the rock-boring sea urchin, *Echinometra mathaei*. Bull. Mar. Sci. 41, 579–584.
57. McClanahan, T. R. & Mwashote, B. M. 1988 Changes in Kenian coral reef community structure and function due to exploitation. Hydrobiologia 166, 269–276.
58. Bak, R. 1990 Patterns of echinoid bioerosion in two Pacific coral reef lagoons. Mar. Ecol. Prog. Ser. 66, 267–272.
59. Mokady, O., Lazar, B. & Loya, Y. 1996 Echinoid bioerosion as a major structuring force of red sea coral reefs. Biol. Bull. 190, 367–372.
60. Mills, S. C., Peyrot-Clausade, M. & France Fontaine, M. 2000 Ingestion and transformation of algal turf by Echinometra mathaei on Tiahura fringing reef (French Polynesia). J. Exp. Mar. Bio. Ecol. 254, 71–84.
61. Carreiro-Silva, M. & McClanahan, T. R. 2001 Echinoid bioerosion and herbivory on Kenyan coral reefs: The role of protection from fishing. J. Exp. Mar. Bio. Ecol. 262, 133–153.
62. Herrera-Escalante, T., López-Pérez, R. A. & Leyte-Morales, G. E. 2005 Bioerosion caused by the sea urchin Diadema mexicanum (Echinodermata: Echinoidea) at Bahías de Huatulco, Western Mexico. Rev. Biol. Trop. 53 Suppl 3, 263–273.
63. Bellwood, D. 1995 Direct estimate of bioerosion by two parrotfish species, Chlorurus gibbus and C. sordidus, on the Great Barrier Reef, Australia. Mar. Biol. 419–429.
64. Ong, L. & Holland, K. N. 2010 Bioerosion of coral reefs by two Hawaiian parrotfishes: species, size differences and fishery implications. *Mar. Biol.* **157**, 1313–1323. (doi:10.1007/s00227-010-1411-y)
65. Lokrantz, J., Nyström, M., Thyresson, M. & Johansson, C. 2008 The non-linear relationship between body size and function in parrotfishes. Coral Reefs 27, 967–974.
66. Bellwood, D. R. & Choat, J. H. 1990 A functional analysis of grazing in parrotfishes (family Scaridae): the ecological implications. *Environ. Biol. Fishes* **28**, 189–214. (doi:10.1007/BF00751035)
67. Hughes, T. P., Bellwood, D. R., Folke, C. S., McCook, L. J. & Pandolfi, J. M. 2007 No-take areas, herbivory and coral reef resilience. *Trends Ecol. Evol.* **22**, 1–3. (doi:10.1016/j.tree.2006.10.009)
68. Pratchett, M. S., Anderson, K. D., Hoogenboom, M. O., Widman, E., Baird, A. H., Pandolfi, J. M., Edmunds, P. J. & Lough, J. M. 2015 Spatial , temporal and taxonomic variation in coral growth - implications for the structure and function of coral reef ecosystems . *Oceanogr. Mar. Biol. an Annu. Rev.* **53**, 215–295.
69. Darling, E. S., Alvarez-Filip, L., Oliver, T. A., McClanahan, T. R., Côté, I. M. & Bellwood, D. 2012 Evaluating life-history strategies of reef corals from species traits. *Ecol. Lett.* **15**, 1378–1386. (doi:10.1111/j.1461-0248.2012.01861.x)
70. Darling, E. S., McClanahan, T. R. & Côté, I. M. 2013 Life histories predict coral community disassembly under multiple stressors. *Glob. Chang. Biol.* **19**, 1930–1940.
71. Perry, C. T., Murphy, G. N., Graham, N. A. J., Wilson, S. K., Januchowski-Hartley, F. A. & East, H. K. 2015 Remote coral reefs can sustain high growth potential and may match future sea-level trends. *Sci. Rep.* **5**, 18289. (doi:10.1038/srep18289)
72. Graham, N. A. J., Jennings, S., Macneil, M. A., Mouillot, D. & Wilson, S. K. 2015 Predicting climate-driven regime shifts versus rebound potential in coral reefs. *Nature* **518**, 94–97. (doi:10.1038/nature14140)
73. McCook, L. J. 1999 Macroalgae , nutrients and phase shifts on coral reefs : scientific issues and managemennt

consequences for the Great Barrier Reef. *Coral Reefs* **18**, 357–367. (10.1007/s003380050213)

74. Porter, J. W. 1972 Patterns of species diversity in Caribbean reef corals. *Ecology* **53**, 745–748. (doi:10.2307/1934796)
75. Madin, J. S. & Connolly, S. R. 2006 Ecological consequences of major hydrodynamic disturbances on coral reefs. *Nature* **444**, 477–480. (doi:10.1038/nature05328)
76. Graham, N. A. J. & Nash, K. L. 2013 The importance of structural complexity in coral reef ecosystems. *Coral Reefs* **32**, 315–326. (doi:10.1007/s00338-012-0984-y)
77. Feary, D. A., Almany, G. R., McCormick, M. I. & Jones, G. P. 2007 Habitat choice, recruitment and the response of coral reef fishes to coral degradation. *Oecologia* **153**, 727–737. (doi:10.1007/s00442-007-0773-4)
78. Wilson, S. K., Graham, N. A. J., Fisher, R., Robinson, J., Nash, K., Chong-Seng, K., Polunin, N. V. C., Aumeeruddy, R. & Quatre, R. 2012 Effect of macroalgal expansion and marine protected areas on coral recovery following a climatic disturbance. *Conserv. Biol.* **26**, 995–1004. (doi:10.1111/j.1523-1739.2012.01926.x)
79. Harris, A., Wilson, S., Graham, N. & Sheppard, C. 2014 Scleractinian coral communities of the inner Seychelles 10 years after the 1998 mortality event. *Aquat. Conserv. Mar. Freshw. Ecosyst.* **24**, 667–679. (doi:10.1002/aqc.2464)
80. Kennedy, E. V et al. 2013 Avoiding coral reef functional collapse requires local and global action. *Curr. Biol.* **23**, 912–918. (doi:10.1016/j.cub.2013.04.020)
81. McClanahan, T. R., Graham, N. A. J., Calnan, J. M. & MacNeil, M. A. 2007 Toward pristine biomass: reef fish recovery in coral reef marine protected areas in Kenya. *Ecol. Appl.* **17**, 1055–1067. (doi: 10.1890/06-1450)
82. Selig, E. R. & Bruno, J. F. 2010 A global analysis of the effectiveness of marine protected areas in preventing coral loss. *PLoS One* **5**, e9278. (doi:10.1371/journal.pone.0009278)
83. Hijmans, R. J., Phillips, S., Leathwick, J. & Elith, J. 2015 dismo: Species Distribution Modeling.
